# Identification of UHRF2 as a novel DNA interstrand crosslink sensor protein

**DOI:** 10.1371/journal.pgen.1007643

**Published:** 2018-10-18

**Authors:** Anna Motnenko, Chih-Chao Liang, Di Yang, David Lopez-Martinez, Yasunaga Yoshikawa, Bao Zhan, Katherine E. Ward, Jiayang Tian, Wilhelm Haas, Paolo Spingardi, Benedikt M. Kessler, Skirmantas Kriaucionis, Steven P. Gygi, Martin A. Cohn

**Affiliations:** 1 Department of Biochemistry, University of Oxford, Oxford, United Kingdom; 2 Department of Cell Biology, Harvard Medical School, Boston, MA, United States of America Medicine, Kitasato University, Aomori, Japan; 3 Ludwig Cancer Research, Nuffield Department of Clinical Medicine, University of Oxford, Oxford, United Kingdom; 4 Target Discovery Institute, Nuffield Department of Medicine, University of Oxford, Oxford, United Kingdom; MRC Laboratory of Molecular Biology, UNITED KINGDOM

## Abstract

The Fanconi Anemia (FA) pathway is important for repairing interstrand crosslinks (ICLs) between the Watson-Crick strands of the DNA double helix. An initial and essential stage in the repair process is the detection of the ICL. Here, we report the identification of UHRF2, a paralogue of UHRF1, as an ICL sensor protein. UHRF2 is recruited to ICLs in the genome within seconds of their appearance. We show that UHRF2 cooperates with UHRF1, to ensure recruitment of FANCD2 to ICLs. A direct protein-protein interaction is formed between UHRF1 and UHRF2, and between either UHRF1 and UHRF2, and FANCD2. Importantly, we demonstrate that the essential monoubiquitination of FANCD2 is stimulated by UHRF1/UHRF2. The stimulation is mediating by a retention of FANCD2 on chromatin, allowing for its monoubiquitination by the FA core complex. Taken together, we uncover a mechanism of ICL sensing by UHRF2, leading to FANCD2 recruitment and retention at ICLs, in turn facilitating activation of FANCD2 by monoubiquitination.

## Author summary

Fanconi Anemia is a genetic disease where patients typically have congenital abnormalities, develop bone marrow failure and suffer from cancer predisposition. The cells in patients have a reduced ability to repair a type of DNA damage where the two strands of the DNA double helix are physically linked together, and this failure in repair is believed to contribute to cause of the disease. Many proteins are involved in repairing this type of DNA damage in healthy individuals, via a complex DNA repair pathway called the Fanconi Anemia pathway. Here, we report the identification of a new player in this pathway. The protein, called UHRF2, is able to sense the DNA damage in the genome, and thereby help to initiate the healthy repair of the damage. In addition to improving our molecular understanding of the Fanconi Anemia pathway, in the long term, this new knowledge could have medical implications for diagnosis and therapy relating to pathologies involving this type of DNA damage.

## Introduction

DNA interstrand crosslinks (ICLs) can obstruct the critical processes of transcription and replication [1]. If unrepaired, an ICL can perturb accurate chromosome segregation during mitosis when replication has not been completed due to the crosslink, this can additionally lead to replication fork collapse causing double strand DNA breaks. For these reasons, repair of ICLs is required to preserve genomic stability. In humans, the Fanconi anemia (FA) pathway is critical to ICL repair. FA is a congenital disorder, which leads to bone marrow failure and predisposition to various cancers. FA patients are sensitive to ICLs due to defects in the FA pathway [2]. At least 22 FA proteins operate together to repair an ICL, assisted by several other key DNA repair factors, which are not known to be bona fide FA proteins [3–5]. The complicated repair process relies on multiple DNA repair pathways coordinated through the FA pathway. The repair process entails recognition of the damage, multiple incisions by nucleases, translesion synthesis (TLS), nucleotide excision repair (NER) and homologous recombination (HR) [6]. A key step in initiation of repair requires recognition of the ICL. We previously identified UHRF1 as an ICL sensor protein [7]. We showed that UHRF1 interacts directly with ICLs *in vitro* and *in vivo* and that its recruitment *in vivo* is functionally important to initiate the repair reaction. UHRF1 has also been shown to function with BRCA1 in the double strand break repair pathway choice [8]. UHRF2, a paralogue of UHRF1, is structurally similar to UHRF1, with five recognizable domains. UHRF2 contains a ubiquitin-like domain (UBL), Tandem-Tudor domain (TTD), Plant Homeodomain (PHD), SET and RING associated domain (SRA), and Really Interesting New Gene domain (RING). The UBL domain resides in the N-terminus of the protein and structurally resembles ubiquitin. This structural similarity is evident when comparing the UBL of UHRF1 to Ubiquitin, as superimposition of the structures yields an RMSD value of 0.52Å [9]. TTD and PHD domains have been shown to work together in the recognition of H3K9me2/3 histone marks [10], while the SRA domain, which is unique to UHRF1 and UHRF2 in humans [11] is a DNA binding domain, shown to recognize hemimethylated DNA [12]. Lastly, the RING domain of UHRF2 is an E3 ligase. UHRF2 has been shown to possess autoubiquitinaton activity and is capable of ubiquitinating PCNP, however, there is not much known about potential other substrates of the UHRF2 E3 ligase [13]. UHRF2 is not very well characterized, however it has been shown to be important in cell cycle progression and can cause G1 phase cell cycle arrest through its interaction with the Cdk2–cyclin E complex [14, 15]. UHRF2 has been implicated in genome maintenance as it has been shown to interact with PCNA through a PIP box motif [16], additionally, UHRF2 was shown to be recruited to DNA damage sites, primarily through its TTD-PHD and SRA domains [17].

Here we report the identification of UHRF2 as a sensor for ICLs in humans. We demonstrate that UHRF2 functionally cooperates with UHRF1 in the ICL repair process, and that both proteins physically interact. Importantly, we show that UHRF1 and UHRF2 interact directly with FANCD2, *in vitro* and *in vivo*, and that UHRF1/UHRF2 facilitate the recruitment and retention of FANCD2 to ICLs, providing a mechanistic insight into how UHRF1 and UHRF2 mediate the early stages of ICL repair.

## Results

### UHRF2 is recruited to ICLs *in vitro* and *in vivo*

**Fig 1:**
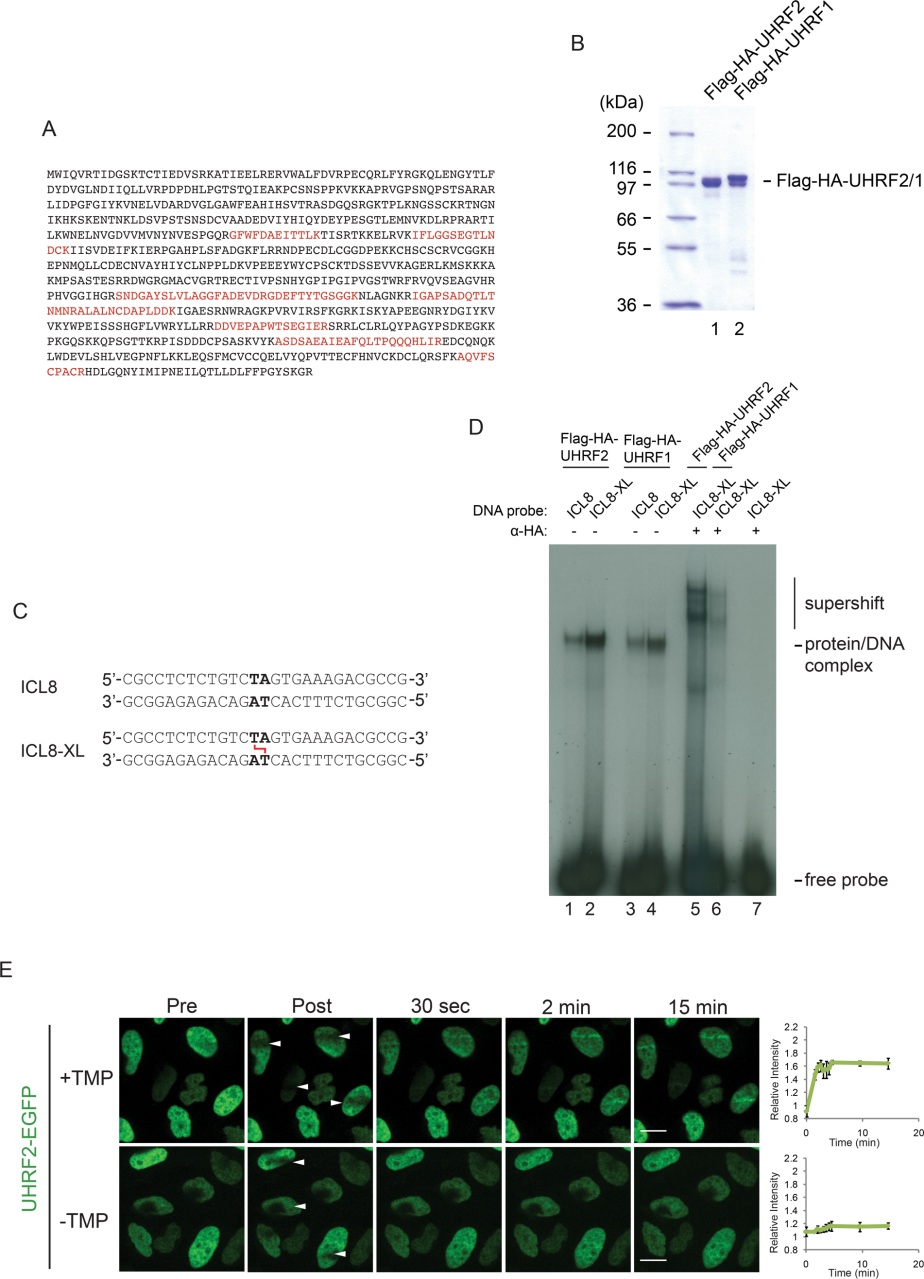
UHRF2 binds ICLs *in vitro* and is recruited to ICLs *in vivo*. A) Mass spectrometry identification of UHRF2. 11 peptides were identified. Amino acids present in identified peptides are indicated in red color. B) Coomassie blue stain of Flag-HA-tagged recombinant UHRF1 and UHRF2 proteins purified from Sf9 insect cells. C) Schematic of non-ICL and ICL-containing DNA probes used for EMSA in (D). D) EMSA showing the preferential binding of recombinant Flag-HA-tagged UHRF2 and UHRF1 to ICL-containing DNA probe (ICL8-XL) compared to control DNA probe (ICL8) DNA. A super-shift using HA antibody against Flag-HA-tagged UHRF2 and UHRF1 confirms that the protein/DNA complex is specifically formed by UHRF2 and UHRF1 binding to the DNA probe. E) Live-cell imaging of HeLa cells expressing exogenous EGFP-tagged UHRF2 with and without pre-treatment with TMP. Cells were micro irradiated at the sites indicated with white arrows. Scale bar indicates 10μm. Charts indicate quantification of relative intensity of signal at the irradiated sites. UHRF2 was recruited only the presence of TMP. Error bars show SEM n = 4/treatment.

We previously devised a purification scheme to identify proteins interacting with ICL-containing DNA [7]. Using this scheme, we excised a band from an SDS-PAGE gel containing such proteins, and identified all proteins in the band by mass spectrometry [7]. We identified a total of two proteins, namely the paralogues UHRF1 and UHRF2, by 76 and 11 peptides, respectively (Fig 1A and [7]). We previously found UHRF1 to interact directly with ICLs both *in vitro* and *in vivo*, therefore we decided to investigate whether UHRF2 might possess similar properties. We expressed full-length UHRF2 in Sf9 insect cells and purified the protein to homogeneity (Fig 1B). We then designed a DNA probe containing a single ICL in the center (Fig 1C), and radiolabeled it by ^32^P. Using electrophoretic mobility shift assay (EMSA) we then assessed the ability of UHRF2 to interact directly with an ICL. As expected, we observed a weak complex formed with the control DNA molecule, and a stronger complex formed with the probe containing a central ICL (Fig 1D, lanes 1–2). In good agreement, we observed the same trend when assaying the DNA-binding properties of UHRF1 (Fig 1D, lanes 3–4). Both the UHRF1 and UHRF2 protein-DNA complexes could be supershifted using specific antibodies, confirming their identities (Fig 1D, lanes 5–6).

Given the clear result *in vitro*, we then sought to test whether UHRF2 is recruited to ICLs *in vivo*. We previously described an experimental system where we can observe the live recruitment of fluorophore-fused proteins to ICLs using live-cell imaging [7]. The system is based on stable expression of the protein of interest as a fusion protein with a fluorophore. Cells are then incubated with the psoralen analog TMP (trimethylpsoralen), and placed in a spinning disc microscope. While cells are being imaged, a local region of the nucleus is irradiated with a laser beam, causing the formation of ICLs in that region. Cells can then be monitored over time, allowing for observation and quantification of recruitment of the protein of interest to ICLs. Using this method, we analyzed the recruitment of UHRF2-EGFP to ICLs in HeLa cells. We observed recruitment of UHRF2-EGFP already 30 seconds after the introduction of the ICLs, kinetics similar to that of UHRF1, and the strength of the signal increased over the following minutes (Fig 1E). Taken together, UHRF2 displays distinct characteristics of an ICL sensor protein, namely direct interaction with ICLs *in vitro* and rapid recruitment to ICLs *in vivo*.

### UHRF2 is functionally important for repair of ICLs

**Fig 2:**
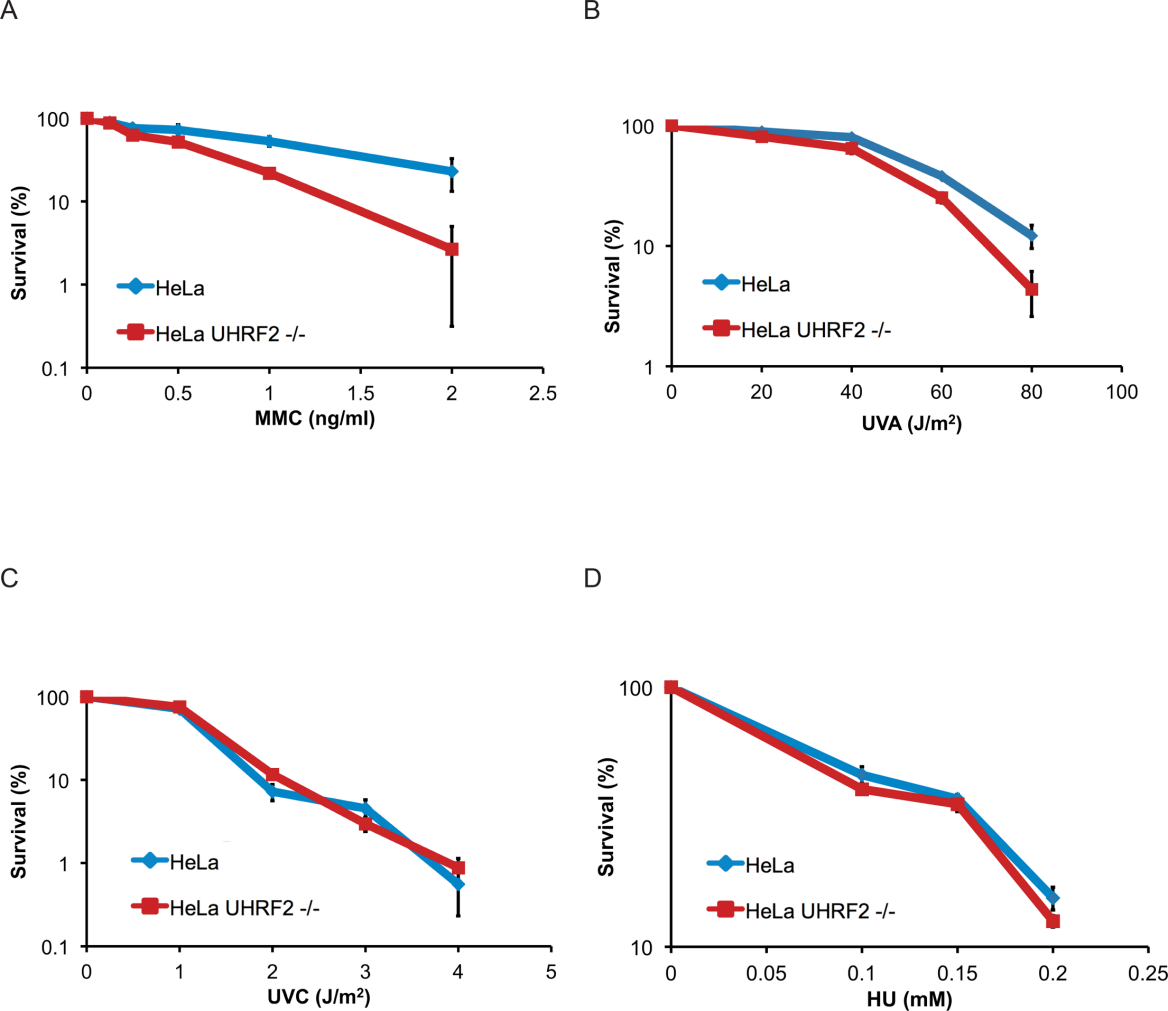
Depletion of UHRF2 reduces cell survival in response to ICL-causing agents. A) Clonogenic survival assay of HeLa and HeLa UHRF2-/- cells in response to MMC. Depletion of UHRF2 reduces cell survival. Experiment was repeated 3 times, each with a p-value <0.01 at 2 ng/ml MMC. B) Clonogenic survival assay of HeLa and HeLa UHRF2 -/- cells in response to TMP/UVA. Depletion of UHRF2 reduces cell survival. Experiment was repeated 2 times, each with a p-value <0.01 at 80 J/m^2^. C) Clonogenic survival assay of HeLa and HeLa UHRF2-/- cells in response to UVC. Experiment was performed once in triplicate. D) Clonogenic survival assay of HeLa and HeLa UHRF2-/- cells in response to HU. Experiment was performed once in triplicate.

Since UHRF2 is recruited to ICLs, we speculated that the protein might be involved in ICL repair. To test this directly, we evaluated the ability of cells to respond to ICLs after depleting the UHRF2 protein. To this end, we disrupted the *UHRF2* gene in HeLa cells using CRISPR/Cas9 genome editing, leading to a complete depletion of endogenous UHRF2 protein (S1A–S1C Fig). HeLa and HeLa UHRF2 -/- cells were then subjected to a clonogenic survival assay, where cells are treated with increasing concentrations of MMC, and survival is assessed by counting the number of colonies formed. As expected, depletion of UHRF2 resulted in sensitivity to MMC (Figs 2A and S2A). We also tested the sensitivity towards ICLs formed by TMP. Again, UHRF2 deficient cells were sensitive compared to control cells (Fig 2B). We assessed the sensitivity of UHRF2 deficient cells to other types of DNA damage and replication stress, and observed no sensitivity towards UVC and HU (hydroxyurea) (Fig 2C and 2D).

### The SRA domain of UHRF2 is important for its recruitment to ICLs

**Fig 3:**
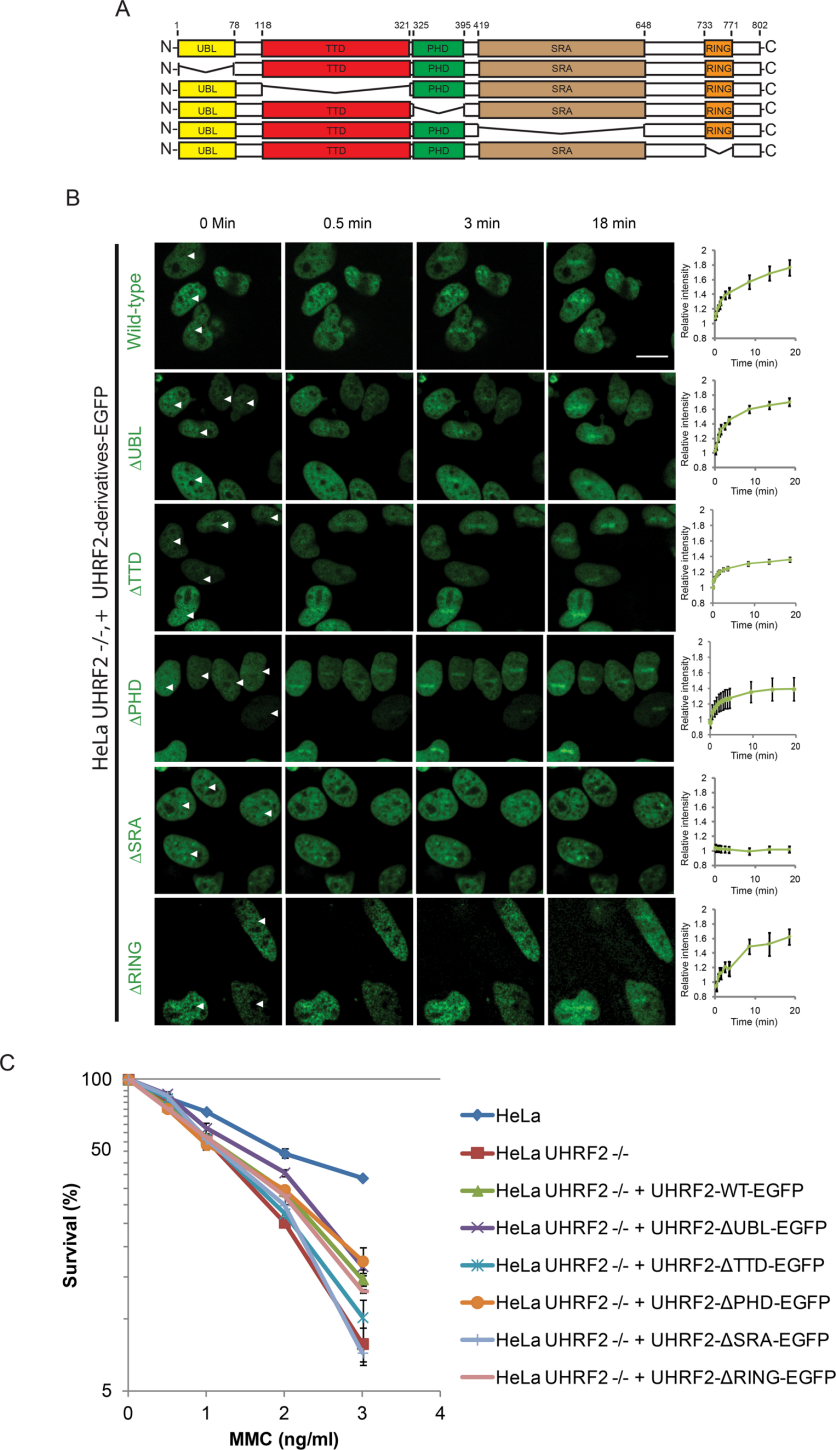
The SRA domain of UHRF2 is required for its recruitment to ICLs. A) Schematic representation of UHRF2 indicating positions of domains and deletions. B) Live-cell imaging of HeLa UHRF2 -/- cells (CRIPR/Cas9-mediated knockout) complemented with EGFP-tagged UHRF2 containing deletions as indicated in (A). Cells were pre-treated with TMP and micro irradiated at the sites indicated with white arrows. Scale bar indicates 10μm. Charts indicate quantification of relative intensity of signal at the irradiated sites. The p-values between EGFP-UHRF2 and EGFP-UHRF2-ΔTTD, EGFP-UHRF2-ΔPHD and EGFP-UHRF2-ΔSRA at the 20 min time-point are 0.001, 0.001, and 0.0000008, respectively. Error bars show SEM n = 8/treatment. C) Clonogenic survival assay of HeLa UHRF2 -/- cells complemented with the UHRF2 domain deletion mutants indicated in (A). Experiment was repeated 2 times and with p-values for EGFP-UHRF2-ΔSRA and EGFP-UHRF2-ΔTTD compared to EGFP-UHRF2 of 0.03 and 0.175, respectively. Error bars show SEM.

UHRF2 contains 5 unique domains, the UBL, PHD, TTD, SRA and RING domains (Fig 3A). To gain more mechanistic insight into how UHRF2 is recruited to ICLs, we decided to abrogate each of the 5 domains one at a time, and then assess the recruitment of the resulting mutant proteins *in vivo* using live-cell imaging. EGFP-tagged versions of UHRF2 containing each of the 5 deletions were then stably expressed in UHRF2 -/- cells (S2B Fig). Wildtype UHRF2-EGFP was recruited normally, showing quick recruitment to ICLs (Fig 3B). Deletion of the UBL, PHD or RING domains caused no or minor reduction of recruitment, while deletion of the TTD domains caused a mild reduction of recruitment (Fig 3B). In contrast, deleting the SRA domain completely abrogated the recruitment to ICLs (Fig 3B).

Since the SRA domain appears critical for recruitment of UHRF2 to ICLs, we next sought to test whether this and the other domains are also functionally important for the ICL repair function of UHRF2. The five deletion versions of UHRF2, where each of the domains were abrogated one at a time, were expressed in UHRF2 -/- cells, and the resulting cell lines were assessed for sensitivity to ICLs. In good agreement with our live-cell imaging data, deletion of the SRA domain completely abrogated the function of UHRF2 in response to ICLs, whereas deletion of the TTD domain caused a mild reduction of function, and deletion of UBL, PHD and RING domains caused no reduction of function (Fig 3C).

### UHRF1 and UHRF2 are required for monoubiquitination of FANCD2 and for its recruitment to ICLs

**Fig 4:**
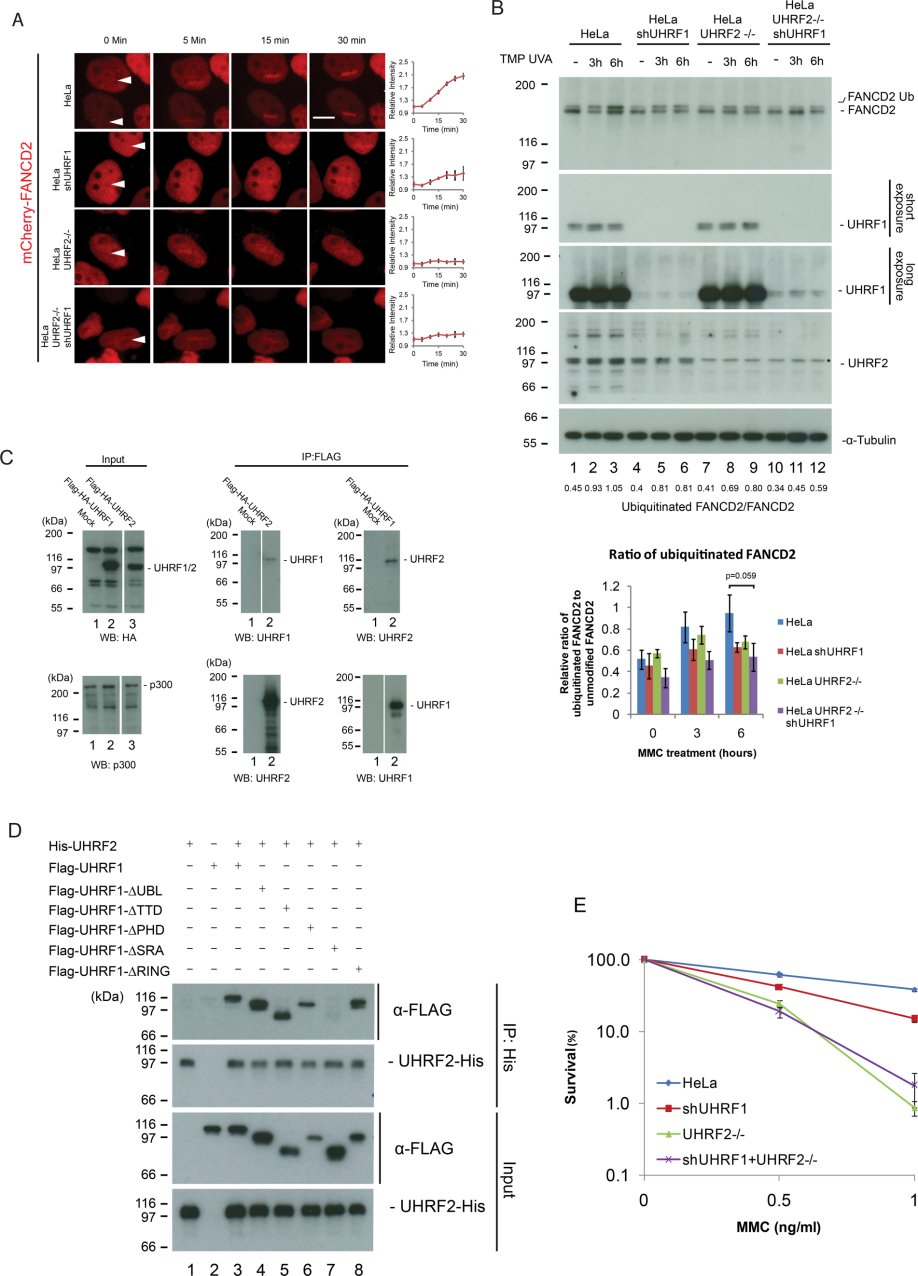
UHRF2 and UHRF1 mediate FANCD2 recruitment and ubiquitination in response to DNA damage. A) HeLa cells expressing mCherry-tagged FANCD2 where subjected to depletion of UHRF1 by shRNA and/or depletion of UHRF2 by CRISPR/Cas9-mediated knockout, pre-treated with TMP, and then microirradiated at the sites indicated with white arrows. Charts indicate quantification of relative intensity of signal at the irradiated sites. Depletion of UHRF1 and UHRF2 impairs FANCD2 recruitment. Scale bar indicates 10μm n = 8/treatment. B) Western blot analysis of lysates from HeLa cells or HeLa cells where UHRF1 and/or UHRF2 were depleted using shRNA-mediated knockdown or CRISPR/Cas9-mediated knockout following treatment with TMP/UVA and harvested at 3 and 6 hours. Asterisk indicate unspecific band. Chart represents data from three independent experiments (two replicate experiments are shown in S5 Fig), and shows ratio of FANCD2-Ub to FANCD2. Strong accumulation of monoubiquitinated FANCD2 (FANCD2-Ub) occurs in HeLa cells but is reduced when UHRF1 and UHRF2 are depleted (p-value = 0.059 for the 6h timepoint). C) Flag-HA-tagged UHRF2 or Flag-HA-tagged UHRF1 were expressed in HeLa cells and immunoprecipitated from nuclear extracts using anti-Flag antibodies. Immunoprecipitates were analyzed by immunoblotting using anti-UHRF2 and anti-UHRF1 antibodies, as indicated. UHRF2 is co-immunoprecipitated with UHRF1 and vice versa. p300 is used as a loading control. D) *In vitro* binding assays of His-UHRF2 and wild type or deletion mutants of Flag-UHRF1 (purified from Sf9 cells). His-UHRF2 was immunoprecipitated and immunoblotting demonstrated that the SRA domain of UHRF2 is responsible for the protein-protein interaction between UHRF1 and UHRF2. E) Clonogenic survival assay of HeLa cells where UHRF1 and/or UHRF2 are depleted. Error bars show SEM. n = 3.

Given the clear functional importance of UHRF2 in ICL repair, and the previously suggested interplay between UHRF1 and FANCD2 [7], we speculated that UHRF2 might function by recruiting FANCD2 to ICLs. To test this directly, we stably expressed mCherry-tagged FANCD2 in HeLa cells and in HeLa UHRF2 -/- cells (S2C Fig), and assessed the ability of mCherry-FANCD2 to be recruited to ICLs *in vivo*. As expected, mCherry-FANCD2 was recruited normally in control cells (Fig 4A). However, the recruitment was reduced when UHRF2 was depleted. We then assessed the recruitment of mCherry-FANCD2 upon UHRF1 reduction using shRNA (S2C Fig), and also observed reduced recruitment of mCherry-FANCD2 to ICLs (Fig 4A). Cellular depletion of UHRF1 and UHRF2 simultaneously led to strong reduction in recruitment of mCherry-FANCD2 (Fig 4A). In good agreement with these data, we observed the same phenotype of reduced FANCD2 recruitment when cellular levels of UHRF1 and UHRF2 were reduced using only shRNA (S3A and S3B Fig).

Recruitment of FANCD2 to ICLs has traditionally been assessed via its formation of nuclear foci. Therefore, to test our conclusions, we asked whether the ability of FANCD2 to form such foci in response to either MMC or TMP/UVA, depends on the activities of UHRF1 and UHRF2. We assessed the FANCD2 foci formation upon depletion of UHRF1, UHRF2, or both. The formation of FANCD2 foci was markedly reduced upon depletion of either or both of the proteins (S3C and S3D Fig), strengthening the conclusions based on our experiments using localized introduction of ICLs (Fig 4A).

Monoubiquitination of FANCD2 on lysine 561 is required for its activation and accumulation on ICLs *in vivo*. Therefore, we hypothesized that UHRF1 and UHRF2 might affect the monoubiquitination of FANCD2. To check this directly, we depleted UHRF1, UHRF2 or both UHRF1 and UHRF2 in HeLa cells (S4A Fig) and determined the ability of the resulting cells to monoubiquitinate FANCD2. We treated the cells with TMP and UVA and monitored the amount of monoubiquitinated FANCD2 over time. We observed a clear increase in the amount of monoubiquitinated FANCD2 after 3 and 6 hours (Fig 4B, lanes 1–3, S4B and S4C Fig). In contrast, monoubiquitination was reduced upon depletion of UHRF1 and/or UHRF2 (Fig 4B, lanes 4–12, S4B and S4C Fig). FACS analysis determined that a change in the population of S-phase cells upon reduction of UHRF1/2 did not cause this phenotype (S4D Fig). On the other hand, an accumulation of G2/M cells in UHRF1/2 depleted cells was observed, a phenotype typically observed in cells with a disrupted FA pathway.

To further test the conclusion that UHRF1/2 stimulates FANCD2 recruitment and monoubiquitination, we assessed the recruitment of FANCD2 to ICLs in cells depleted of endogenous UHRF1, and complemented with a mutant of UHRF1 where the SRA domain has been deleted. This mutant protein of UHRF1 is not recruited to ICLs itself. In these cells, the recruitment of FANCD2 is abrogated, underscoring a functional relationship between UHRF1/2 and FANCD2 recruitment (S5A Fig).

Both UHRF1 and UHRF2 are E3 ligases. It could therefore be a possibility that these enzymes directly monoubiquitinate FANCD2. To test this possibility, we assessed the monoubiquitination of FANCD2 *in vitro*, using purified components. While FANCL, the E3 ligase for FANCD2, provided robust monoubiquitination of FANCD2, neither of the UHRF1 and UHRF2 enzymes possessed such activity *in vitro*, suggesting that these enzymes are not E3 ligases for FANCD2 (S5B–S5E Fig).

### UHRF1 and UHRF2 form a protein-protein interaction

Given the observed contribution by both UHRF1 and UHRF2 towards recruitment of FANCD2, we speculated that the two proteins might form a physical interaction not described in the literature. To test this directly, we expressed either Flag-HA-UHRF1 or Flag-HA-UHRF2 proteins in HeLa cells, and assessed the co-immunoprecipitation of the two proteins with endogenous UHRF2 and UHRF1, respectively, post TMP/UVA treatment. As predicted, immunoprecipitates of Flag-HA-UHRF1 contained UHRF2, and immunoprecipitates of Flag-HA-UHRF2 contained UHRF1 (Fig 4C). To further characterize the interaction between these two proteins we turned to *in vitro* co-immunoprecipitation, utilizing purified recombinant UHRF1 domain deletion mutants and purified recombinant UHRF2. We identified the domain of UHRF1 that is responsible for the interaction with UHRF2 as the SRA domain (Figs 4D and S5F).

The interaction between UHRF1 and UHRF2 prompted us to determine whether this translates to a functional genetic relationship between the two genes. We depleted UHRF1 and UHRF2 by shRNA and CRISPR/Cas9, respectively (S4A Fig). Survival of the resulting cell lines in response to MMC was subsequently assessed. We observed a sensitivity resulting from reduction of UHRF1 and UHRF2 by knockdown or knockout, respectively, as expected. Importantly, in good agreement with the biochemical data, we did not observe a significant further sensitization of UHRF2 -/- cells upon depleting UHRF1 by knockdown (Fig 4E).

**Fig 5:**
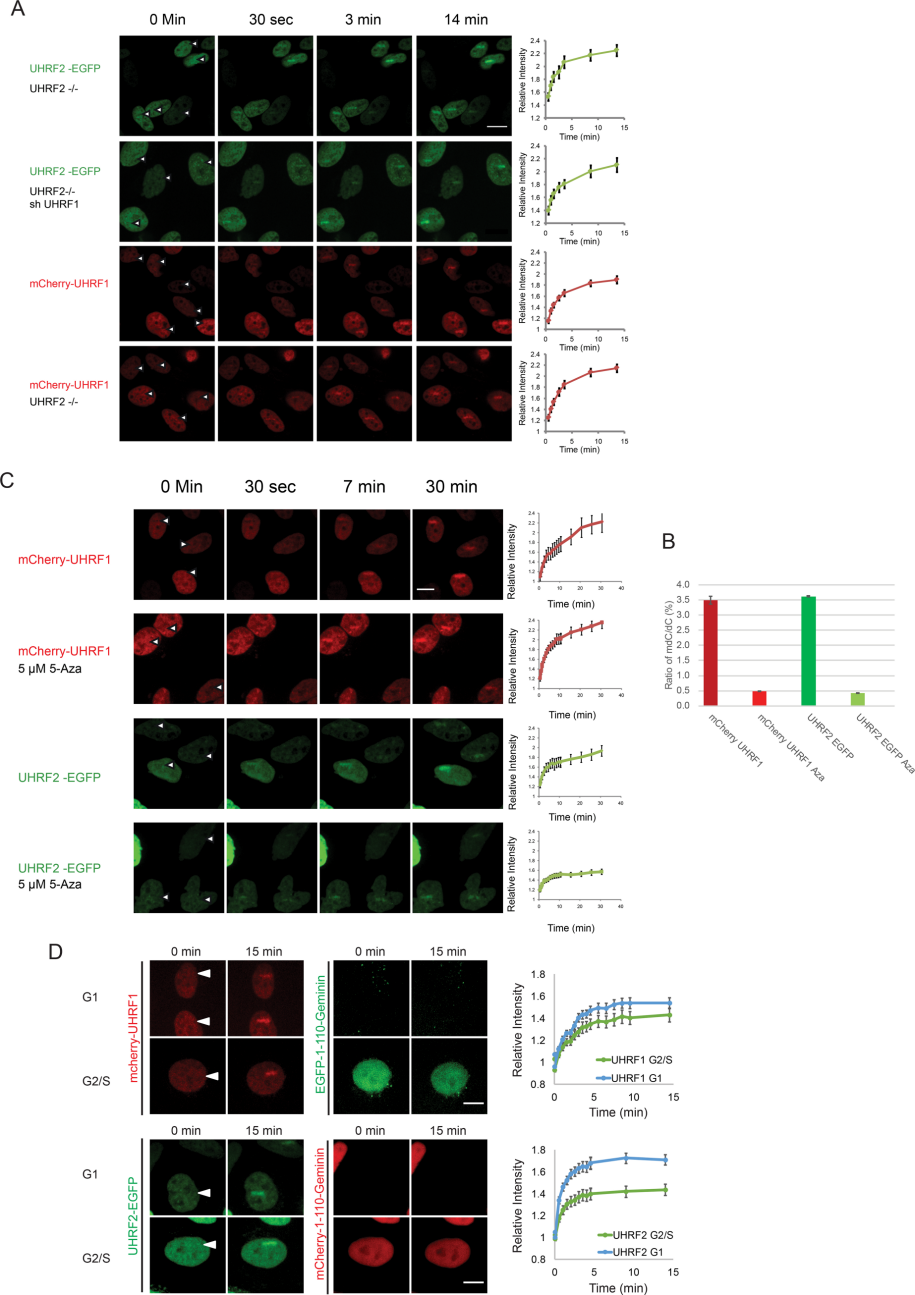
A) UHRF1 and UHRF2 are recruited to ICLs *in vivo* independently of each other. HeLa cells expressing either EGFP-tagged UHRF2 or mCherry-tagged UHRF1, were depleted of endogenous UHRF2 (CRISPR/Cas-9) and/or UHRF1 (shRNA). The cells were pre-treated with TMP and then microirradiated at the indicated sites. Charts indicate quantification of relative intensity of signal at the irradiated sites. Error bars show SEM n = 8/treatment. B) Genomic DNA from cells in (C) was assessed for the degree of DNA methylation. The percent of methylated cytosines vs. non-methylated cytosines is shown for each treatment. C) Cells expressing mCherry-tagged UHRF1 or EGFP-tagged UHRF2 were subjected to 5 μM 5-Aza-2'-deoxycytidine for 5 days. Cells were pre-treated with TMP and microirradiated at the sites indicated by the white arrows. The charts show the quantification of the stripe intensity. Error bars show SEM, n = 8 per treatment. The p-value for UHRF2 Aza compared to control UHRF2 is 0.005. D) Cells expressing either UHRF1-mCherry and EGFP-1-110-Geminin, or, UHRF2-EGFP and mCherry-1-110-Geminin, were imaged for 15 after the introduction of ICLs by microirradiation following treatment with TMP. The intensity of the fluorescent proteins in the microirradiated areas were quantified and plotted, in either G1 or S/G2-phase cells. Representative images shown. Error is shown as SEM, n = 16 per fluorophore. Scale bar indicates 10μm.

These data suggest that both UHRF1 and UHRF2 contribute towards FANCD2 recruitment and towards ICL repair. However, it is not clear whether these two repair factors affect the recruitment of each other or whether their simultaneous recruitment is needed for full FANCD2 recruitment. To differentiate between these two possibilities, we assessed the recruitment of UHRF1 to ICLs in the absence of UHRF2, and *vice versa*. Depleting either UHRF1 or UHRF2 did not significantly reduce the recruitment of UHRF2 and UHRF1, respectively (Fig 5A). Additionally, over-expression of mCherry-UHRF1 in HeLa UHRF2 -/- cells, did not restore monoubiquitination of FANCD2 (S6A and S6B Fig). Taken together, these data show that UHRF1 and UHRF2 both need to be recruited to ICLs for subsequent FANCD2 recruitment and monoubiquitination to take place, and that the recruitment of either factor takes place independently of the other factor.

### UHRF1 and UHRF2 are recruited to ICLs independently of DNA methylation in all stages of the cell cycle

Both UHRF1 and UHRF2 have been reported to interact with hemimethylated DNA [12]. It was therefore plausible that such an interaction could play a role in the recruitment of these proteins to ICLs. We decided to test this hypothesis directly. Cells expressing either mCherry-UHRF1 or UHRF2-EGFP were treated with 5-Aza-2'-deoxycytidine for 5 days to reduce DNA methylation. The treatment led to a near elimination of DNA methylation (Fig 5B). The ability of the proteins to be recruited to ICLs was then assessed and compared to untreated cells. We observed no significant reduction in recruitment of UHRF1 to ICLs when DNA methylation was reduced, demonstrating that recruitment to ICLs does not depend on DNA methylation, while the recruitment of UHRF2 was slightly reduced. (Fig 5C).

To further strengthen this conclusion, we assessed the degree of UHRF1 and UHRF2 recruitment to ICLs in the G1- and S-phases of the cell cycle. The amount of hemimethylated DNA is significant in the S-phase, where replication leads to synthesis of new non-methylated DNA, thereby creating hemimethylated DNA. On the other hand, G1 cells contain much less hemimethylated DNA [18]. We stably expressed mCherry-UHRF1 or UHRF2-EGFP together with a cell cycle marker containing the 110 N-terminal amino acids of Geminin fused with EGFP or mCherry, respectively. The cell cycle marker is absent in G1-cells and present in S/G2-cells [19]. Using these stable cell lines, we could then assess the recruitment of UHRF1 and UHRF2 in G1 and S/G2 cells without the use of any perturbing drugs, which could affect the cells. We observed strong recruitment of both UHRF1 and UHRF2 in both G1 and S/G2 cells, with UHRF2 recruitment of slightly higher amplitude in G1 cells compared to S/G2 cells (Fig 5D). Perhaps a larger fraction of UHRF2 is mobile in G1 compared to G2/S. A previous report suggested that UHRF2 is active in G1 as a cell cycle regulator [20]. The kinetics of recruitment for both proteins was not dependent on the cell cycle.

### UHRF1 and UHRF2 form a direct protein-protein interaction with FANCD2

**Fig 6:**
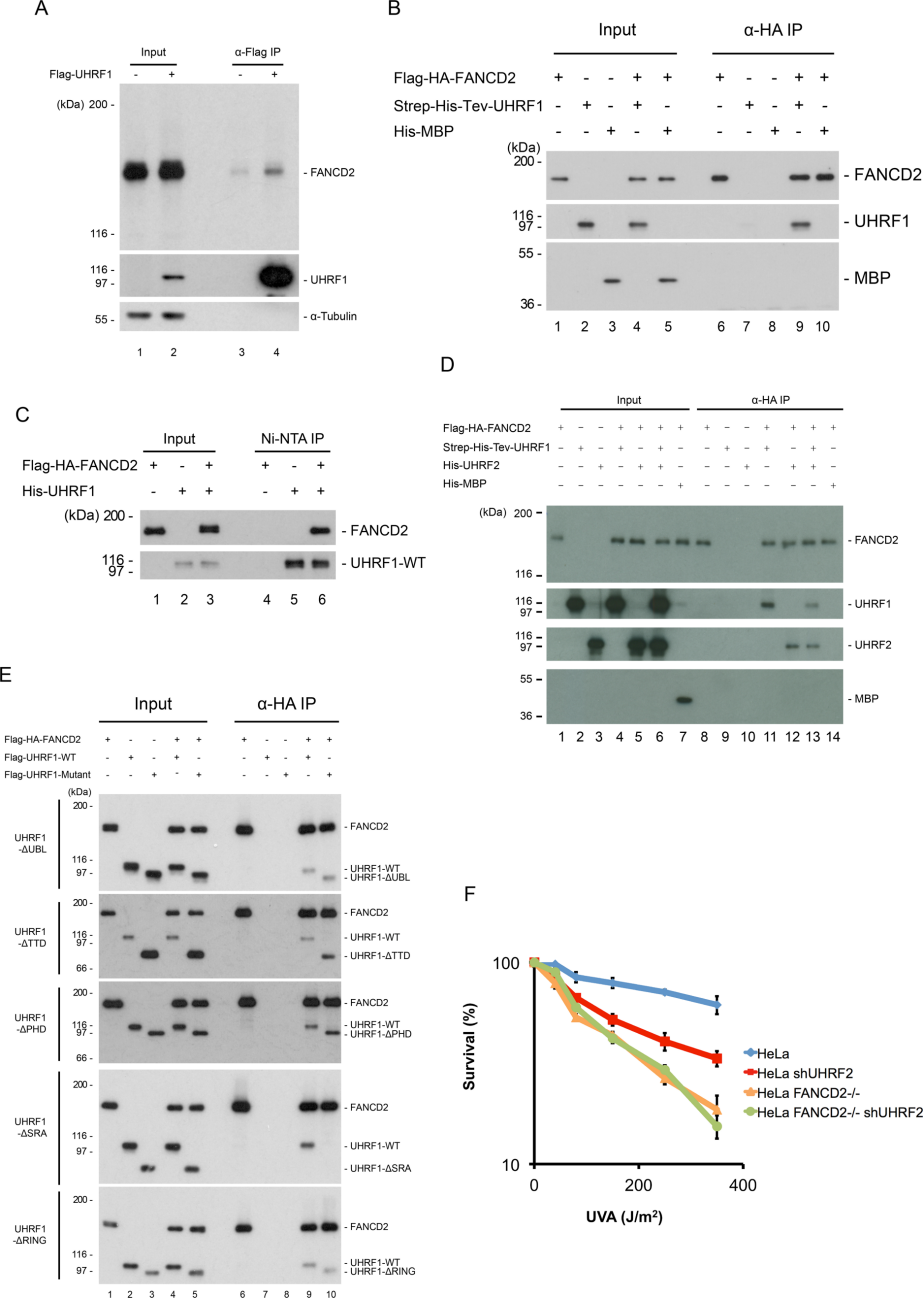
UHRF1 interacts directly with FANCD2. A) Immunoprecipitation of Flag-UHRF1 from HeLa.shUHRF1 cells expressing exogenous Flag-UHRF1. HeLa.Scramble was used as a negative control. Cells were treated with TMP/UVA and allowed to recover for 1 hour before lysis, immunoprecipitation and immunoblotting. B) *In vitro* binding assay using Flag-HA-FANCD2, Strep-6xHis-Tev-UHRF1 (both purified from Sf9 cells) and 6xHis-MBP (purified from *E*. *coli*). HA-FANCD2 was immunoprecipitated and immunoblotting demonstrated co-immunoprecipitation of UHRF1 but not of MBP, which was used as a negative control. C) *In vitro* binding assay using Flag-HA-FANCD2 and 6xHis-UHRF1 (both purified from Sf9 cells). 6xHis-UHRF1 was purified using NTA-agarose, and immunoblotting demonstrated co-purification of FANCD2. D) *In vitro* binding assay using HA-FANCD2, Strep-6xHis-Tev-UHRF1, 6xHis-UHRF2 (all purified from Sf9 cells) and 6xHis-MBP (purified from *E*. *coli*). HA-FANCD2 was immunoprecipitated and immunoblotting demonstrated co-immunoprecipitation of UHRF1 and UHRF2 but not of MBP, which was used as a negative control. E) *In vitro* binding assays of Flag-HA-FANCD2 and wild type or deletion mutants of Flag-UHRF1 (purified from Sf9 cells). HA-FANCD2 was immunoprecipitated and immunoblotting demonstrated that the SRA domain of UHRF1 is responsible for the protein-protein interaction between UHRF1 and FANCD2. F) Clonogenic survival assay of HeLa, HeLa FANCD2 -/-, HeLa shUHRF2, and HeLa FANCD2 -/- shUHRF1 cells in response to TMP/UVA. Experiment was performed once in triplicate.

UHRF1 and UHRF2 are recruited to the ICL shortly after DNA damage and this is, in turn, necessary for the recruitment of FANCD2 to the ICL. We also show that depletion of UHRF1 and UHRF2 reduces the degree of FANCD2 monoubiquitination. It was recently found that monoubiquitination of FANCD2 occurs after its recruitment to DNA [21]. Therefore, it is possible that UHRF1 and UHRF2 facilitate the retention of FANCD2 at the ICL, potentially through a direct protein-protein interaction. To test this, we first assessed whether the two proteins interact with each other *in vivo*. Cellular extracts from HeLa cells expressing either epitope-tagged UHRF1, or epitope-tagged FANCD2, were subjected to immunoprecipitation after ICLs had been induced by TMP/UVA. Western blot analysis confirmed the presence of endogenous FANCD2 in the UHRF1 immunoprecipitate (Fig 6A, lane 4) and endogenous UHRF1 in the FANCD2 immunoprecipitate (S7A Fig, lane 4). These data suggest that some FANCD2 interact with UHRF1 in the cell.

We then went on to test whether the UHRF1 and FANCD2 interact directly. To this end, we purified both proteins in their full-length forms from Sf9 cells, and assessed their ability to interact *in vitro*. We observed a direct and strong interaction between FANCD2 and UHRF1 (Fig 6B, lane 9). Importantly, there was no interaction between FANCD2 and MBP, used as a negative control, confirming the specificity of the interaction (Fig 6B, lane 10). We confirmed the observed interaction by the reciprocal purification (Fig 6C, lane 6). To additionally reinforce the interaction data, we analyzed either UHRF1 or FANCD2 alone, or after they had been incubated together, by gel filtration. These experiments also confirmed a UHRF1/FANCD2 protein-protein interaction (S7B Fig). Additional experiments demonstrated that UHRF2 interacts with FANCD2 in a similar fashion (Fig 6D).

Given that FANCD2 is likely to be monoubiquitinated before or shortly after its recruitment to the ICL, we wanted to test whether the ubiquitination status affects its interaction with UHRF1. We purified either unmodified or monoubiquitinated FANCD2 to homogeneity from HeLa cells, and assessed their abilities to interact with UHRF1. Both forms interacted equally well with UHRF1, suggesting that the interaction is not dependent on monoubiquitination of FANCD2 (S7C Fig).

Our data demonstrate that UHRF1 and FANCD2 form a direct protein-protein interaction. We next sought to determine which domain of UHRF1 is interacting with FANCD2. To this end, we expressed and purified mutant versions of UHRF1 from Sf9 cells, each containing either the UBL, TTD, PHD, SRA or RING domains deleted (S7D Fig). Again, a strong interaction was observed between wild type UHRF1 and FANCD2 (Fig 6E, lane 10). Likewise, all mutant proteins where either the UBL, TTD, PHD or RING domain was deleted interacted well with FANCD2, whereas deletion of the PHD domain might slightly enhance the binding. However, when we deleted the SRA domain it completely abolished the interaction with FANCD2 (Fig 6E, lane 10). These data suggest that the function of UHRF1/2 in ICL repair might depend on FANCD2. To test this possibility, we depleted UHRF2 in cells already depleted of FANCD2, and asked whether we would observe further sensitization. We observed no further sensitization, suggesting that UHRF2 indeed functions together with FANCD2 in ICL repair (Figs 6F and S7E).

## Discussion

The early stages of ICL repair are critical. Recognition of the ICL leading to the subsequent deployment of essential repair factors is a key event for successful and accurate repair, and is still poorly understood.

### UHRF2 is a new ICL sensor protein

Here we show that UHRF2, a paralogue of UHRF1, plays an essential role in the initial stage of the ICL repair process. Simultaneous depletion of UHRF1 and UHRF2 significantly sensitizes cells to ICL forming agents, such as MMC and TMP/UVA. We found that UHRF2 interacts directly with an ICL *in vitro*, and that it is recruited to ICLs within seconds of their appearance *in vivo*, using live-cell imaging. We also found that both UHRF1 and UHRF2 are needed for efficient recruitment of FANCD2 to ICLs, and for its subsequent monoubiquitination by the FA core complex.

UHRF1 and UHRF2 are relatively homologous, with an overall protein sequence identity of 54% and overall sequence similarity of 68%. However, the two proteins are not redundant for their cellular ICL repair functions, instead we found the two proteins to functionally cooperate in ICL repair. Recruitment of UHRF1 and UHRF2 to ICLs in cells occurs independently. However, recruitment of both proteins is required for normal FANCD2 recruitment and monoubiquitination. We show that FANCD2 is not monoubiquitinated by UHRF1 and UHRF2, however it is possible that other ICL repair proteins are ubiquitinated by these E3 ligases after recruitment to the ICL. As both UHRF1 and UHRF2 are E3 ligases and have been shown to be important for chromatin remodeling, it is also possible that UHRF1/2 mediate histone modification at the site of the ICL, or recruit the DNA methyl transferase DNMT1 mediating DNA methylation [11]. Additionally, UHRF2 has been shown to directly interact with PCNA, perhaps facilitating PCNA recruitment for subsequent DNA repair [16, 20] and UHRF1 has been reported to interact with nucleases involved in ICL repair [22]. The various proposed roles of UHRF1 in ICL repair has been discussed extensively elsewhere [4].

### UHRF1 and UHRF2 interact directly with FANCD2 via the SRA domain

We have uncovered a direct protein-protein interaction between UHRF1/UHRF2 and FANCD2. Interestingly, the SRA domain of UHRF1, which is only present in UHRF1 and UHRF2 in humans [11], is required for this interaction. The SRA domains share 75% amino acid identity. The structure of the SRA domain of UHRF1 in complex with hemimethylated DNA has been solved [23–25] as have the structures of the SRA domain of UHRF2 in complex with either hemimethylated or hemihydroxymethylated DNA [12]. In all cases, the protein is shaped as a saddle sitting on the DNA, creating a large surface all around the SRA domain, available for interaction with other proteins, such as FANCD2. Intriguingly, it was similarly shown that DNMT1 also interacts with the SRA domain, important for its recruitment to hemimetylated DNA [26, 27]. We found that while recruitment of UHRF2 and UHRF1 to ICLs occur independently of each other, both proteins need to be present at the ICL for full repair. It is possible that a stronger retention of FANCD2 on chromatin is obtained when both proteins are present. Future structural studies will be required to understand the atomic nature of the ICL/UHRF1/UHRF2/FANCD2 interactions. We found that DNA methylation is not a main prerequisite for UHRF1 and UHRF2 recruitment to ICLs.

### UHRF2 and UHRF1 act to retain FANCD2 on DNA allowing for its monoubiquitination

**Fig 7:**
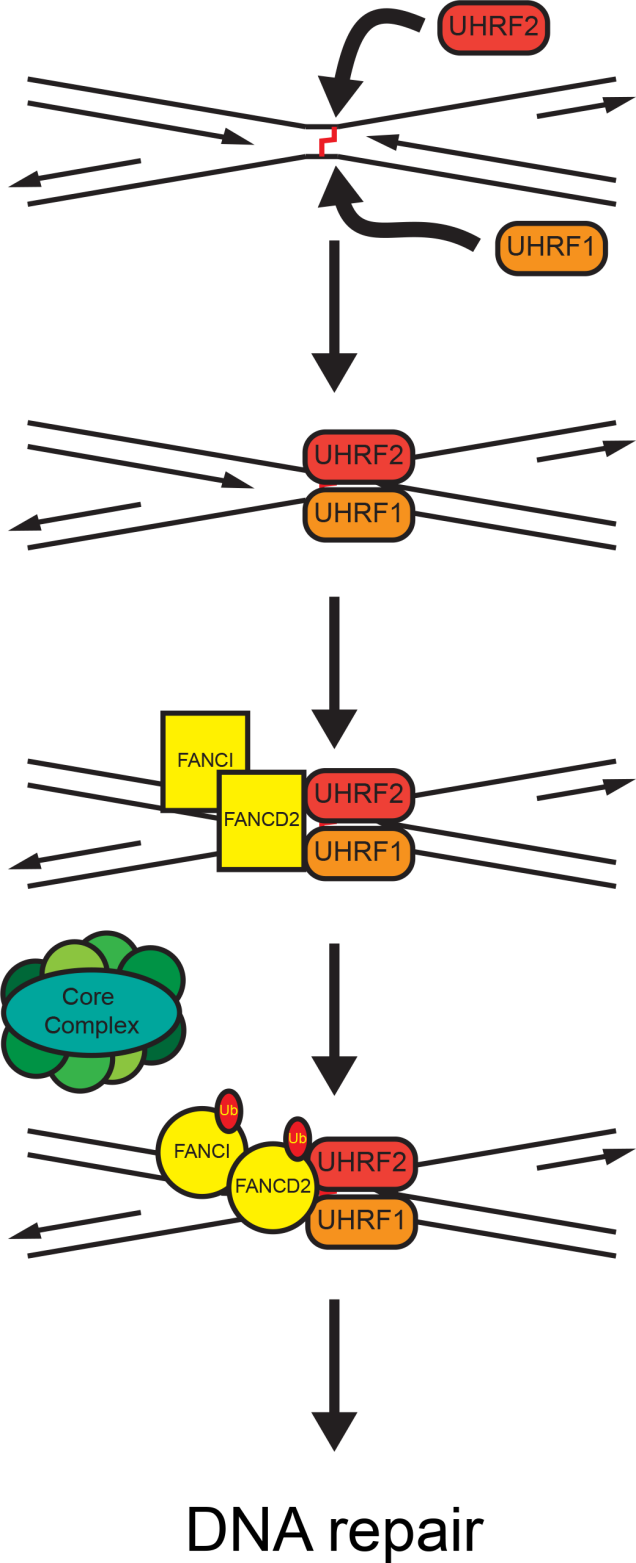
A proposed model of FANCD2 and FANCI recruitment and activation at ICLs via UHRF1 and UHRF2. UHRF1 and UHRF2 are rapidly and independently recruited to the site of the ICL. Recruitment of UHRF1 and UHRF2 facilitate recruitment/retention of the FANCD2/FANCI complex. When FANCD2 and FANCI are bound at the ICL a putative conformational change allows for monoubiquitination by the core complex. The now active FANCD2/FANCI complex activates downstream repair proteins, leading to repair of the ICL.

Our data support a model where UHRF1/2 are recruited very quickly to the ICL, facilitating the following recruitment, and importantly, retention, of FANCD2. If UHRF1 and UHRF2 function to retain monoubiquitinated FANCD2 on chromatin, we would expect some monoubiquitination to take place in the absence of UHRF1/UHRF2, but we would expect little or no retention of FANCD2 at ICLs, measured by live-cell imaging. This is indeed what we observe. Also, in good agreement, we recently demonstrated that FANCD2 is ubiquitinated after its recruitment to DNA as opposed to before recruitment [21]. Therefore, it is plausible that retention of FANCD2 at the ICL via a direct interaction with UHRF1/UHRF2, allows for the ICL repair to initiate (Fig 7). We speculate that UHRF1 and UHRF2 form a type of landing platform for FANCD2 on DNA, ensuring the necessary retention of FANCD2 permitting for its monoubiquitination and activation preventing its dissociation from DNA. It is plausible that the FA core complex is recruited independently by the FANCM/FAAP24 complex [28]. Interestingly, we observe recruitment of UHRF1/UHRF2 to ICLs both in the S- and G1-phases of the cell cycle. It is known that FANCD2 is not recruited efficiently in G1. We speculate that UHRF1/UHRF2 interact with and mark ICLs during G1 allowing for prompt recruitment and monoubiquitination of FANCD2 as soon as the cell enters S-phase. We previously showed that FANCD2 is recruited to ICLs before it is monoubiquitinated, and that monoubiquitination stabilizes its retention on DNA. In good agreement with these data, a non-ubiquitinatable mutant of FANCD2 (K561R) is only slightly enriched at ICLs [21]. Current *in vivo* single-molecule live-cell imaging of these molecules at the millisecond timescale might allow us to test these hypotheses. A recent report placed UHRF1 functionally together with the majority of FA and other ICL repair proteins, further underscoring a role in these processes [29].

### Conclusion

In conclusion, we report UHRF2 as a novel ICL sensor protein. UHRF2 interacts directly with ICLs and facilitates the recruitment and retention of FANCD2 to ICLs. Retention of FANCD2 at the ICL allows for its monoubiquitination, and for the ICL repair process to initiate. It will be interesting to explore whether UHRF1 and UHRF2 also play roles in other DNA repair pathways.

## Materials and methods

### Cell lines, antibodies and plasmids

HeLa and HEK293T cells were grown in DMEM (D5796, Sigma) supplemented with 2.5–10% FBS. Antibodies used were as follows: anti-UHRF1 antibody (sc-373, Santa Cruz Biotechnology); anti-FANCD2 (sc-20022, Santa Cruz Biotechnology); anti-α-Tubulin (5829, Millipore); anti-UHRF2 (sc-54252, Santa Cruz Biotechnology); anti-p300 (sc-584, Santa Cruz Biotechnology) and anti-HA (mouse monoclonal antibody clone 12CA5). EGFP-fused FANCD2 and mCherry-fused UHRF1 cDNA were expressed using a derivative of the pOZ-N plasmid [30]. EGFP-fused UHRF2 cDNA was expressed using a derivative of the pOZ-C plasmid. shRNA-mediated knockdown of the UHRF1, UHRF2, and FANCD2 genes was achieved by expressing the target sequences 5’-AGATATAACGTTAGGGTTT-3’, 5'-TGTAAAGGCTGGTGAAAGA-3' and 5’-GAGCAAAGCCACTGAGGTA-3’, respectively, in the pSuper.retro vector (Clontech). Transfections of plasmid DNA were carried out using FuGENE6 (Promega) according to the manufacturer’s instructions. The UHRF1 domain deletion plasmids were generated as above. The deleted regions of UHRF1 are amino acids 14–91 for the UBL domain, 146–296 for the TTD domain, 323–379 for the PHD domain, 427–630 for the SRA domain and 727–806 for the RING domain. The deleted regions of UHRF2 are amino acids 1–78 for the UBL domain, 118–312 for the TTD domain, 419–648 for the PHD domain, 419–648 for the SRA domain and 733–771 for the RING domain.

### CRISPR-Cas9 gene editing

HeLa UHRF2 -/- cells were generated using plasmid pX459 (Addgene #48139)[31]. The targeting sequence used in the sgRNA was: 5’-GTGCCCGTCTTATTGATCC-3’. Primers, 5’- caccgGTGCCCGTCTTATTGATCC -3’ and 5’- aaacGGATCAATAAGACGGGCACc -3’, were annealed and introduced into the pX459 plasmid through its BbsI site. HeLa cells were transfected with 2 μg of the resulting pX459 plasmid and selected with 4 μg/ml puromycin after 24h. After another 24h cells were plated at low density and clones were picked after 2 weeks. Clones were analyzed using immunoblot analysis and genotyped by sequencing the gRNA target site of the gene locus.

### Preparation of interstrand crosslinked DNA substrates

ICLs were essentially prepared as described [7]. In brief, the DNA oligos were annealed in the buffer containing 10mM Tris-HCl pH7.5, 100mM NaCl and 1mM EDTA. 4,5′,8-trimethylpsoralen (TMP, Sigma, T6137)/UVA (365nm) crosslinking induction was described previously [32]. Interstrand crosslink was confirmed by 8M urea 20% denaturing polyacrylmide gel electrophoresis.

### Mass spectrometric analysis

Proteins were reduced with DTT, cysteine residues were derivatized with iodoacetamide, and the proteins were separated by SDS-PAGE. Proteins from silver stained gel bands were in-gel digested with trypsin [33]. The generated peptide mixtures were subjected to LC-MS/MS using a hybrid linear ion trap/ FT-ICR mass spectrometer (LTQ FT, Thermo Electron) essentially as described previously [34]. MS/MS spectra were assigned by searching them with the SEQUEST algorithm [35] against the human International Protein Index sequence database.

### Protein purification

Proteins purified from Sf9 cells were expressed using the pFastBac1 vector (Life Technologies) with an engineered N-terminal Flag-HA tag. Cell pellets were resuspended in lysis buffer (20mM Tris- HCl pH 8.0, 0.1M KCl, 10% glycerol, 0.1% Tween-20, 2mM β-ME and 0.2mM PMSF). Lysates were clarified by centrifugation, and the supernatants were incubated with M2 anti-Flag agarose resin for 2 hr. The resin was washed extensively, and the protein was eluted in the same buffer containing 0.5mg/ml Flag peptide, but excluding Tween-20. Flag-HA-FANCD2 and Flag-HA-FANCD2-Ub were purified as described [36]. 6xHis-MBP was purified from BL21(DE3) cells using the pET28a vector (plasmid kindly provided by Dr. Mark Howarth) following standard purification methods. E1, E2 and E3 enzymes were purified as described [21].

### *In vitro* protein binding assay

The recombinant proteins were expressed and purified from Sf9 insect cells as indicated above. 1μg of each protein was mixed in the reaction buffer containing 10μg BSA (NEB), 20mM Tris-HCl pH 8.0, 150mM KCl, 10% Glycerol, 2mM β-ME and 0.2mM PMSF in 10μl. The mixture was first incubated at 37°C for 1h for protein complex formation. Protein A sepharose coupled with anti-HA IgG were added subsequently, and the mixture was incubated at 4°C with gentle mixing for 30 minutes. The mixture was then transferred to Micro Bio-Spin Chromatography Columns (Bio-Rad), and washed with the reaction buffer supplemented with 0.1% Tween-20. The proteins were eluted in a buffer contained 100mM Tris-HCl pH 6.8, 100mM KCl, 0.1% Tween 20, 0.2mM EDTA, 10% glycerol and 0.5 mg/ml HA peptide (Sigma).

### Co-immunoprecipitation

HeLa.Scramble, HeLa.shUHRF1 expressing Flag-tagged UHRF1 or HeLa.shFANCD2 expressing Flag-tagged FANCD2 cells were treated with TMP/UVA as described. Cell pellets were incubated with Buffer A (0.1% Triton X-100, 20mM Hepes pH7.9, 5mM MgCl_2_, 10% Glycerol, 1unit/μl Benzonase and 2.5mg/ml DSP (D3669, Sigma)) for 30 minutes on ice, and the Tris pH 8.0 was added to the mixture to 0.2M to quench DSP. 10 times pellet volume of Buffer B (0.15% Triton X-100, 20mM Tris-HCl pH 8.0, 5mM MgCl_2_, 10% Glycerol, 300mM KCl and 0.2mM PMSF) was added to the mixture and incubated for 10 minutes for extraction. Lysates were clarified by centrifugation, and supernatant was used for immunoprecipitation. M2 agarose beads was added to the lysates, and incubated for 2 hours. The resin was washed extensively, and eluted with 0.5mg/ml Flag peptide.

### Electrophoretic mobility shift assay (EMSA)

EMSA was performed as previously described [37] with the following modifications: The binding reaction that contained 1 μg of UHRF1 or UHRF2 and 1 nM radiolabeled DNA, was performed in 10μl containing 25mM Tris-HCl pH 8.0, 100mM NaCl, 6% glycerol, 1mM dithiothreitol (DTT), 5ng poly(dI·dC)-poly(dI·dC)and 1μg bovine serum albumin (BSA, New England Biolabs). For super-shift 2μg anti-HA antibody was added.

### Clonogenic assay

Cells (250–4,000) were plated in 6-well plates and treated with different dosages of the indicated damaging agents on the next day. For TMP/UVA treatment, the cells were treated with 50ng/ml 4,5′,8-trimethylpsoralen (TMP) for 30 minutes, and irradiated with the UVA dosages indicated. Colony formation was scored after 10–14 days using 1% (w/v) crystal violet in methanol.

### Preparation of whole cell lysate

Cells were scraped off the dishes, and centrifuged at 1,000 rpm for 5 minutes. Cell pellets were resuspended and incubated in equal volume of Benzonase buffer (2mM MgCl_2_, 20mM Tris pH 8.0, 10% glycerol, 1% Triton X-100 and 12.5units/ml Benzonase (E1014, Sigma) on ice for 10 minutes. The cells were then lysed by the addition of an equal volume of 2% SDS to reach a final concentration of 1%. Samples were heated at 70°C for 2 minutes. The protein concentration was determined by Bradford assay (Bio-Rad Life Science).

### Live-cell imaging

EGFP-fused FANCD2, EGFP-fused UHRF2, and mCherry-fused UHRF1 cDNA were inserted into the pOZ vector as described above. Live-cell imaging was carried out with an OLYMPUS IX81 microscope connected to PerkinElmer UltraView Vox spinning disk system equipped with a Plan-Apochromat 60x/1.4 oil objective using Volocity software 6.3 for image capturing. EGFP and mCherry were excited with 488 nm and 561 nm laser lines, respectively. Throughout the experiment, these cells were maintained at 5% CO_2_, and 37°C using a live cell environmental chamber (Tokai hit). Confocal image series were typically recorded with a frame size of 512x512 pixels and a pixel size of 139 nm. For localized DNA damage induction, cells were seeded in glass bottom dish (MatTek) and sensitized by incubation in DMEM supplemented with 2.5% FBS and 20 μg/ml 4,5′,8-trimethylpsoralen (TMP) for 30 min at 37°C. Microirradiation was performed using the FRAP preview mode of the Volocity software by scanning (each irradiation time was 100 ms) a preselected area (50x3 pixels) within the nucleus 20–75 times with a 405nm laser set to 100% laser power. The mCherry and EGFP intensities at microirradiated sites were quantified using ImageJ with Fiji, and normalized by their intensities before microirradiation.

### DNA nucleoside analysis by mass spectrometry (HPLC–QQQ)

1 μg of DNA in 200μl of water was added to 200μl of hydrolysis solution (100mM NaCl, 20mM MgCl2, 20mM Tris pH 7.9, 1000U/ml Benzonase, 600mU/ml Phosphodiesterase I, 80 U/ml Alkaline phosphatase, 36 μg/ml EHNA hydrochloride, 2.7mM deferoxamine). The mixture was incubated for two hours and then lyophylised by SpeedVac. The lyophylisate was resuspended in 1000μl of buffer A and 300μl was transferred into an LC-MS vial for analysis. A sample 100 times more dilute was prepared by dilution 5 μl of the original sample into 495 μl of Buffer A.

For the analysis by HPLC–QQQ mass spectrometry, a 1290 Infinity UHPLC was fitted with a Zorbax Eclipse plus C18 column, (1.8μm, 2.1mm 15mm; Agilent) and coupled to a 6495a Triple Quadrupole mass spectrometer (Agilent Technologies) equipped with a Jetstream ESI-AJS source. The data were acquired in dMRM mode using positive electrospray ionisation (ESI1). The AJS ESI settings were as follows: drying gas temperature 230°C, the drying gas flow 14 lmin^-1^, nebulizer 20 psi, sheath gas temperature 400°C, sheath gas flow 11 lmin^-1^,Vcap 2,000 V and nozzle voltage 0 V. The iFunnel parameters were as follows: high pressure RF 110 V, low pressure RF 80 V. The fragmentor of the QQQ mass spectrometer was set to 380 V and the delta EMV set to +200. The gradient used to elute the nucleosides started by a 5-min isocratic gradient composed with 100% bufferA (10 mM ammonium acetate, pH 6) and 0% buffer B (composed of 40% CH3CN) with a flow rate of 0.400 ml min^-1^ and was followed by the subsequent steps: 5–8 min, 94.4% A; 8–9 min, 94.4% A; 9–16min 86.3% A; 16–17 min 0% A; 17–21 min 0% A; 21–24.3 min 100% A; 24.3–25min 100%A. The gradient was followed by a 5min post time to re-equilibrate the column. The raw mass spectrometry data was analysed using the MassHunter Quant Software package (Agilent Technologies, version B.07.01). The transitions and retention times used for the characterization of nucleosides and their adducts are summarized in S1 Table. For the identification of compounds, raw mass spectrometry data was processed using the dMRM extraction function in the MassHunter software. For each nucleoside, precursor ions corresponding to the M-H^+^ and M-Na^+^ species were extracted, and the average of the signal observed from each target ion weighted by response was used for quantification. To utilise the linear range of for each nucleoside, the quantifications of dC, dG and dA were carried out with the diluted samples and quantification of dT and mdC was carried out with the concentrated sample.

## Supporting information

S1 Fig (Data relating to Figs 2 and 3). Establishment of the HeLa UHRF2 -/- cell line A) Schematic representation of UHRF2 truncation at the CRISPR/Cas9 gRNA target site. B) The genomic region at the gRNA target site was PCR amplified and sequenced. Four different allele sequences were detected at the site compared to the wild-type genomic sequence. Below the genomic DNA sequences are shown the amino acid sequences and sites of early stop codons, all truncations occur at the start of the TTD. C) Western blot showing UHRF2 -/- compared to wild-type from HeLa. The UHRF2 antibody displays a non-specific band immediately above the UHRF2 band. The possibility of cellular expression of a peptide containing the N-terminal 127–129 amino acids cannot be tested due to the unavailability of an antibody recognizing this region.
(PDF)

S2 Fig (Data relating to Figs 2 and 3). Expression of UHRF2 deletion variants in HeLa -/- cells. A) Clonogenic survival assay of HeLa cells, UHRF2 -/- and HeLa cells with shRNA mediated UHRF2 knockdown. Cells are sensitized to MMC when UHRF2 is depleted. Error bars represent SEM. B) Expression of EGFP-tagged UHRF2 and derivatives in UHRF2-/- HeLa cells. UHRF2 -/- HeLa cells were stably transfected with EGFP-tagged wild-type UHRF2 and the various UHRF2 domain deletion mutants as indicated. C) Western blot analysis of HeLa cells stably expressing mCherry-tagged FANCD2, in which UHRF1 and/or UHRF2 were depleted by shRNA or CRISPR/Cas9-mediated knockout, respectively. These cell lines were used in the experiments shown in Fig 4A. Asterisk represents a non-specific band.
(PDF)

S3 Fig (Data relating to Fig 4). Recruitment and foci of FANCD2 in response to DNA damage. A) HeLa cells expressing EGFP-tagged FANCD2 where subjected to depletion of UHRF1 and UHRF2 by shRNA, or a Scramble shRNA as control, pre-treated with TMP, and then microirradiatedat the sites indicated with white arrows. Charts indicate quantification of relative intensity of signal at the irradiated sites. Depletion of UHRF1 and UHRF2 reduces FANCD2 recruitment. Scale bar indicates 10μm. Error bars show SEM, n = 5/treatment. B) Western blot analysis of cells used in (A). C) Depletion of UHRF1 and UHRF2 impairs FANCD2 foci formation. HeLa cells cells expressing mCherry-tagged FANCD2 were subjected to shRNA depletion of UHRF1, CRISPR/Cas9 depletion of UHRF2 or both. The cells were pre-treated with TMP and irradiated by UVA or treated with MMC. After 6 hours the cells were counted and the foci counts in the nuclei were quantified in multiple fields of view. Cells with >10 foci/nucleus were considered positive. The percent of positive cells as compared to total cells counted is represented in the chart below. The numbers of cells analyzed for HeLa, HeLa shUHRF1, HeLa UHRF2 -/-, and HeLa UHRF2 -/- shUHRF1, respectively, are 767, 597, 773, 535 for the Control condition, 796, 450, 787, 766 for the MMC condition, and 625, 550, 702, 812 for the TMP/UVA condition. Error bars show mean ±SD of n = 3 independent experiments. Statistical significance is indicated in each case for HeLa versus double knockdown/knockout (t test). * p<0.05, ** p<0.01, *** p<0.001. D) Cells used in microscopy experiment in (C) were harvested were harvested and subjected to immunoblot analysis using the indicated antibodies.
(PDF)

S4 Fig (Data relating to Fig 4). UHRF1 and UHRF2 are required for normal activation and recruitment of FANCD2. A) Western blot analysis of lysates from HeLa cells or HeLa cells where UHRF1 and/or UHRF2 were depleted using shRNA-mediated knockdown or CRISPR/Cas9-mediated knockout. B) and C) Western blot analysis of lysates from HeLa cells or HeLa cells where UHRF1 and/or UHRF2 were depleted using shRNA-mediated knockdown or CRISPR/Cas9-mediated knockout following treatment with TMP/UVA and harvested at 3 and 6 hours. Strong accumulation of monoubiquitinated FANCD2 (FANCD2-Ub) occurs in HeLa cells but is reduced when UHRF1 and UHRF2 are depleted. Replicates used for quantification in Fig 4B. D) FACS analysis of cell lines used in Fig 4B and 4E. Depletion of UHRF1 or UHRF2 does not impact the cell cycle distribution.
(PDF)

S5 Fig (Data relating to Fig 4). UHRF1 and UHRF2 are not E3 ligases for FANCD2. A) HeLa cells expressing EGFP-tagged FANCD2 and shRNA resistant mCherry-UHRF1 with and without the SRA domain where subjected to depletion of by shRNA, pre-treated with TMP, and then microirradiated at the sites indicated with white arrows. Charts indicate quantification of relative intensity of signal at the irradiated sites. Disruption of the SRA domain greatly reduced both UHRF1 and FANCD2 recruitment. Scale bar indicates 10μm. Error bars show SEM, n = 3/treatment. B) *In vitro* ubiquitination assay of FANCD2. Individual components were purified from Sf9 insect cells. FANCL (E3 ligase) supports robust ubiquitinatination of FANCD2. Replacement of FANCL by UHRF1 or UHRF2 does not support FANCD2 monoubiquitination. Switching the E2 ligase UBE2T with UBCH5c (C5) does not allow ubiquitination of FANCD2. C) Western blot of ubiquitination reactions in (B) using an anti-His antibody. The ubiquitin in the ubiquitination reaction is His tagged and is visualized. D) and E) Coomassie blue stain of recombinant proteins used in B. F) Coomassie blue stain of recombinant His-UHRF2, and Flag-tagged wild type and deletion mutants of UHRF1 purified from Sf9 cells, which were used in the experiments presented in Fig 4D.
(PDF)

S6 Fig (Data relating to Figs 4 and 5). Both UHRF1 and UHRF2 are required for FANCD2 monoubiquitination, and they are not redundant. A) Western blot analysis of lysates from HeLa cells or HeLa cells where UHRF2 was depleted using shRNA-mediated knockdown or CRISPR/Cas9-mediated knockout, or mCherry tagged UHRF1 was expressed exogenously, following treatment with TMP/UVA and harvested at 3 and 6 hours. Strong accumulation of monoubiquitinated FANCD2 (FANCD2-Ub) occurs in HeLa cells but is significantly reduced when UHRF2 is depleted and cannot be rescued by UHRF1 over-expression. Asterisk indicates unspecific band. B) Chart shows ratio of FANCD2-Ub to FANCD2.
(PDF)

S7 Fig (Data relating to Fig 6). UHRF1 interacts directly with FANCD2. A) Immunoprecipitation of Flag-FANCD2 from HeLaS3 cells where endogenous FANCD2 was depleted by shRNA. HeLaS3 was used as a negative control. Cells were treated with TMP/UVA and allowed to recover for 1 hour before lysis, immunoprecipitation and immunoblotting. B) Size exclusion chromatography of FANCD2 (top), UHRF1 (middle) or FANCD2 incubated with UHRF1 *in vitro* (bottom). Recombinant proteins were purified from Sf9 cells. Proteins were analyzed on a Superdex 200 5/150 GL chromatography column. C) *In vitro* binding assay of HA-FANCD2, monoubiquitinated HA-FANCD2 (Ub-HA-FANCD2), both purified from HeLa cells, and 6xHis-UHRF1 purified from Sf9 cells. UHRF1 co-immunoprecipitates equally well with HA-FANCD2 and Ub-HA-FANCD2, showing that FANCD2 interacts with UHRF1 independently of monoubiquitination. D) Coomassie blue stain of recombinant Flag-HA-tagged FANCD2, Flag-tagged wild type and deletion mutants UHRF1 and 6xHis-tagged UHRF1 purified from Sf9 cells, which were used in the experiments presented in Fig 6. E) Coomassie blue stain of recombinant Flag-HA-tagged FANCD2, 6xHis-Strep-tagged UHRF1, and 6xHis-tagged UHRF2 purified from Sf9 cells and 6xHis tagged MBP purified form *E*. *coli*, which were used in the experiment presented in Fig 6D. F) Cells used for the clonogenic survival assay experiment shown in Fig 6F were harvested and subjected to immunoblot analysis using the indicated antibodies.
(PDF)

S1 Table DNA nucleoside analysis by mass spectrometry. (PDF)
