# Supplementary figures and images for: Identification of UHRF2 as a novel DNA interstrand crosslink sensor protein
Source: PLoS Genet. 2018 Oct 18;14(10):e1007643. doi: 10.1371/journal.pgen.1007643 (PMC6193622; doi:10.1371/journal.pgen.1007643)

Figure S1

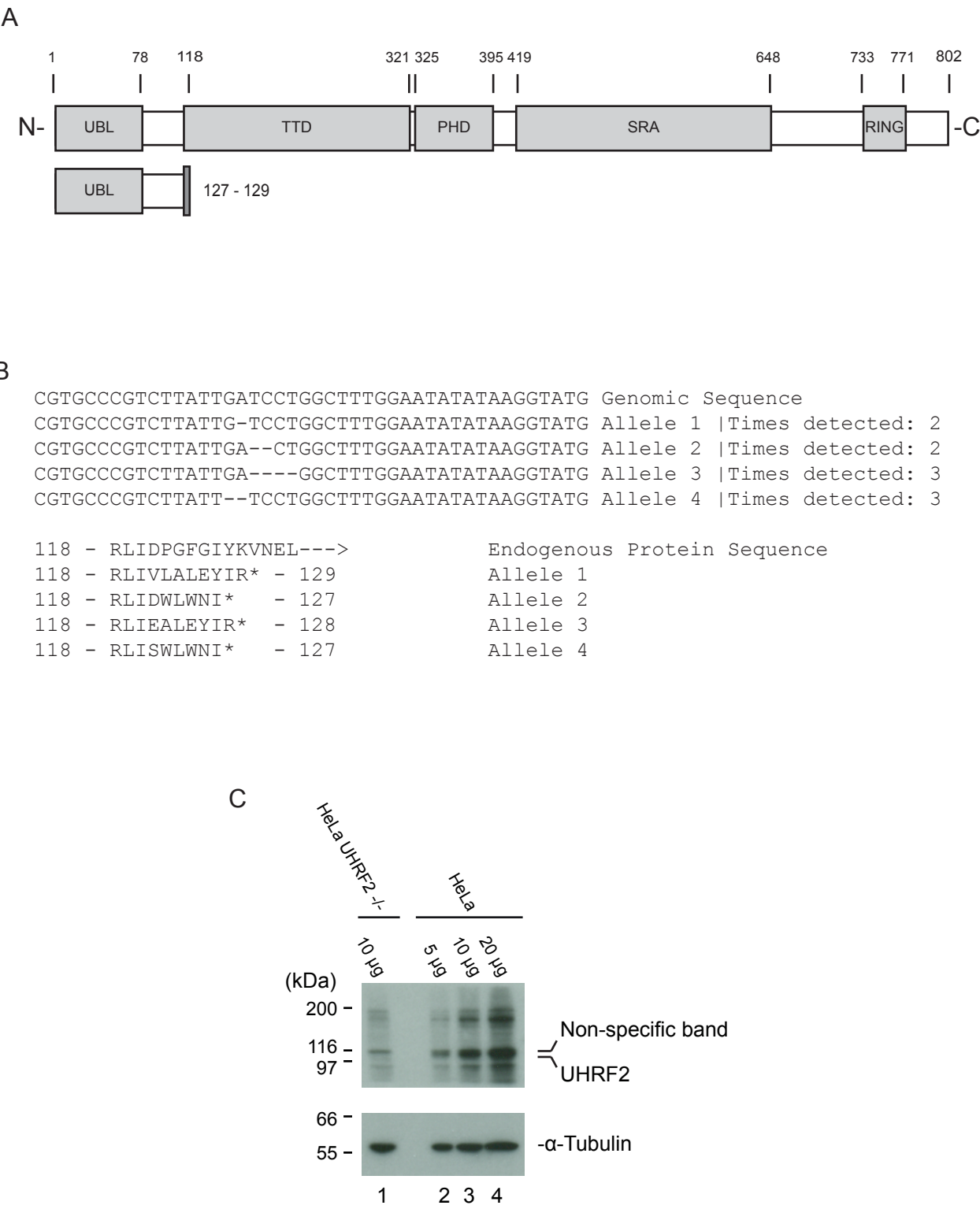

Supplement: S1 Fig — Establishment of the HeLa UHRF2 -/- cell line A) Schematic representation of UHRF2 truncation at the CRISPR/Cas9 gRNA target site. B) The genomic region at the gRNA target site was PCR amplified and sequenced. Four different allele sequences were detected at the site compared to the wild-type genomic sequence. Below the genomic DNA sequences are shown the amino acid sequences and sites of early stop codons, all truncations occur at the start of the TTD. C) Western blot showing UHRF2 -/- compared to wild-type from HeLa. The UHRF2 antibody displays a non-specific band immediately above the UHRF2 band. The possibility of cellular expression of a peptide containing the N-terminal 127–129 amino acids cannot be tested due to the unavailability of an antibody recognizing this region. (PDF) [file pgen.1007643.s001.pdf]

Figure S2

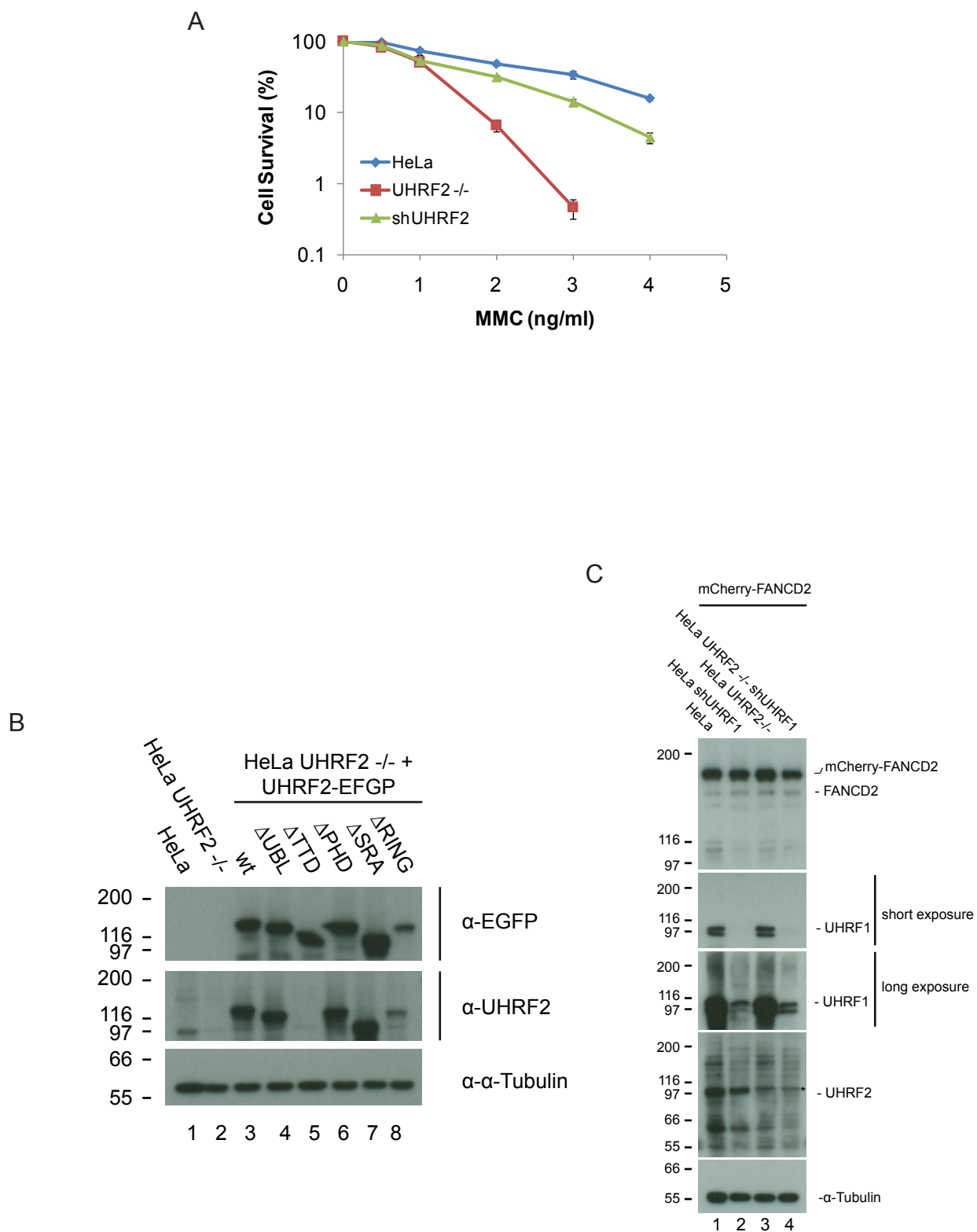

Supplement: S2 Fig — Expression of UHRF2 deletion variants in HeLa -/- cells. A) Clonogenic survival assay of HeLa cells, UHRF2 -/- and HeLa cells with shRNA mediated UHRF2 knockdown. Cells are sensitized to MMC when UHRF2 is depleted. Error bars represent SEM. B) Expression of EGFP-tagged UHRF2 and derivatives in UHRF2-/- HeLa cells. UHRF2 -/- HeLa cells were stably transfected with EGFP-tagged wild-type UHRF2 and the various UHRF2 domain deletion mutants as indicated. C) Western blot analysis of HeLa cells stably expressing mCherry-tagged FANCD2, in which UHRF1 and/or UHRF2 were depleted by shRNA or CRISPR/Cas9-mediated knockout, respectively. These cell lines were used in the experiments shown in Fig 4A. Asterisk represents a non-specific band. (PDF) [file pgen.1007643.s002.pdf]

Figure S3

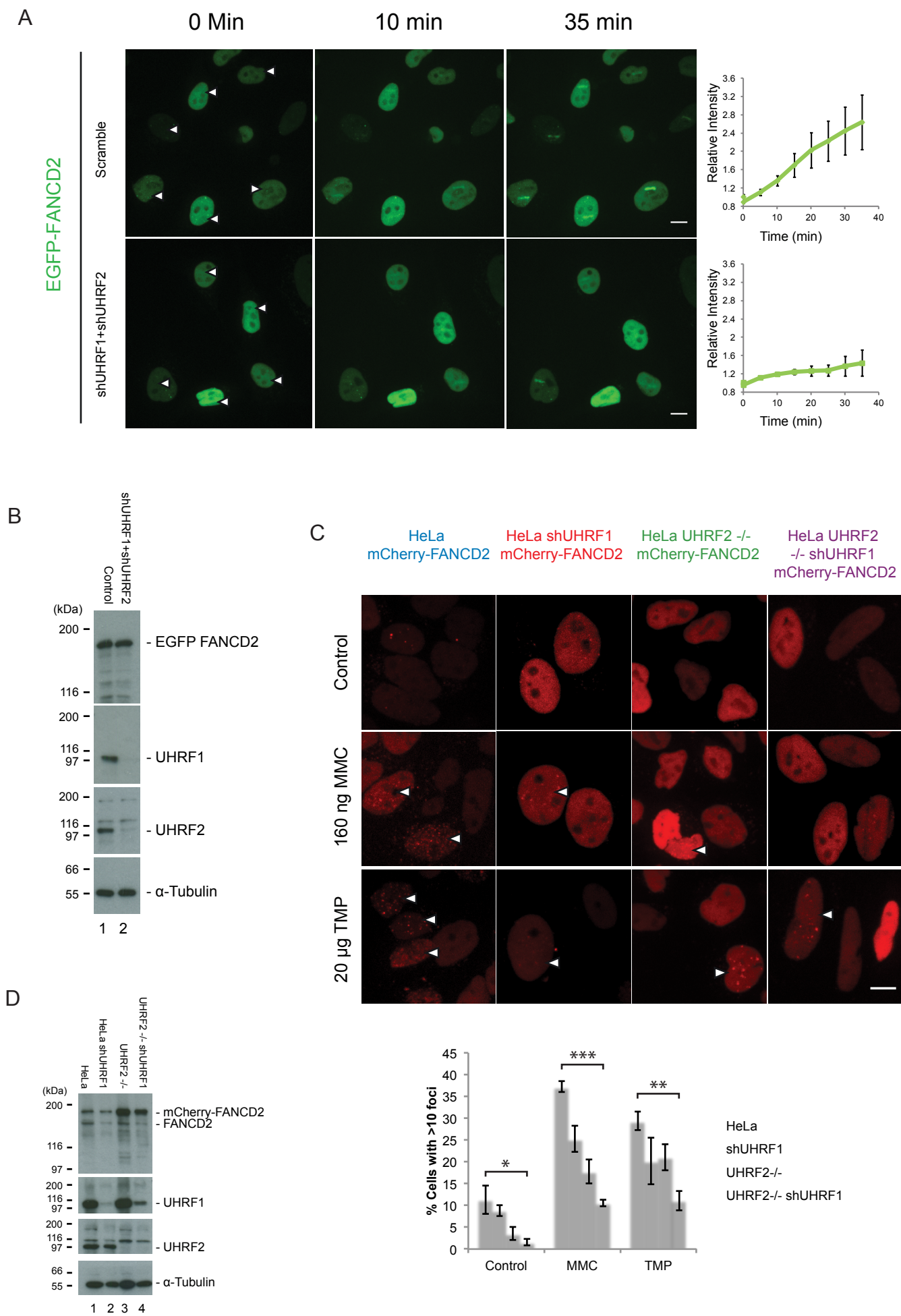

Supplement: S3 Fig — Recruitment and foci of FANCD2 in response to DNA damage. A) HeLa cells expressing EGFP-tagged FANCD2 where subjected to depletion of UHRF1 and UHRF2 by shRNA, or a Scramble shRNA as control, pre-treated with TMP, and then microirradiatedat the sites indicated with white arrows. Charts indicate quantification of relative intensity of signal at the irradiated sites. Depletion of UHRF1 and UHRF2 reduces FANCD2 recruitment. Scale bar indicates 10μm. Error bars show SEM, n = 5/treatment. B) Western blot analysis of cells used in (A). C) Depletion of UHRF1 and UHRF2 impairs FANCD2 foci formation. HeLa cells cells expressing mCherry-tagged FANCD2 were subjected to shRNA depletion of UHRF1, CRISPR/Cas9 depletion of UHRF2 or both. The cells were pre-treated with TMP and irradiated by UVA or treated with MMC. After 6 hours the cells were counted and the foci counts in the nuclei were quantified in multiple fields of view. Cells with >10 foci/nucleus were considered positive. The percent of positive cells as compared to total cells counted is represented in the chart below. The numbers of cells analyzed for HeLa, HeLa shUHRF1, HeLa UHRF2 -/-, and HeLa UHRF2 -/- shUHRF1, respectively, are 767, 597, 773, 535 for the Control condition, 796, 450, 787, 766 for the MMC condition, and 625, 550, 702, 812 for the TMP/UVA condition. Error bars show mean ±SD of n = 3 independent experiments. Statistical significance is indicated in each case for HeLa versus double knockdown/knockout (t test). * p<0.05, ** p<0.01, *** p<0.001. D) Cells used in microscopy experiment in (C) were harvested were harvested and subjected to immunoblot analysis using the indicated antibodies. (PDF) [file pgen.1007643.s003.pdf]

Figure S4

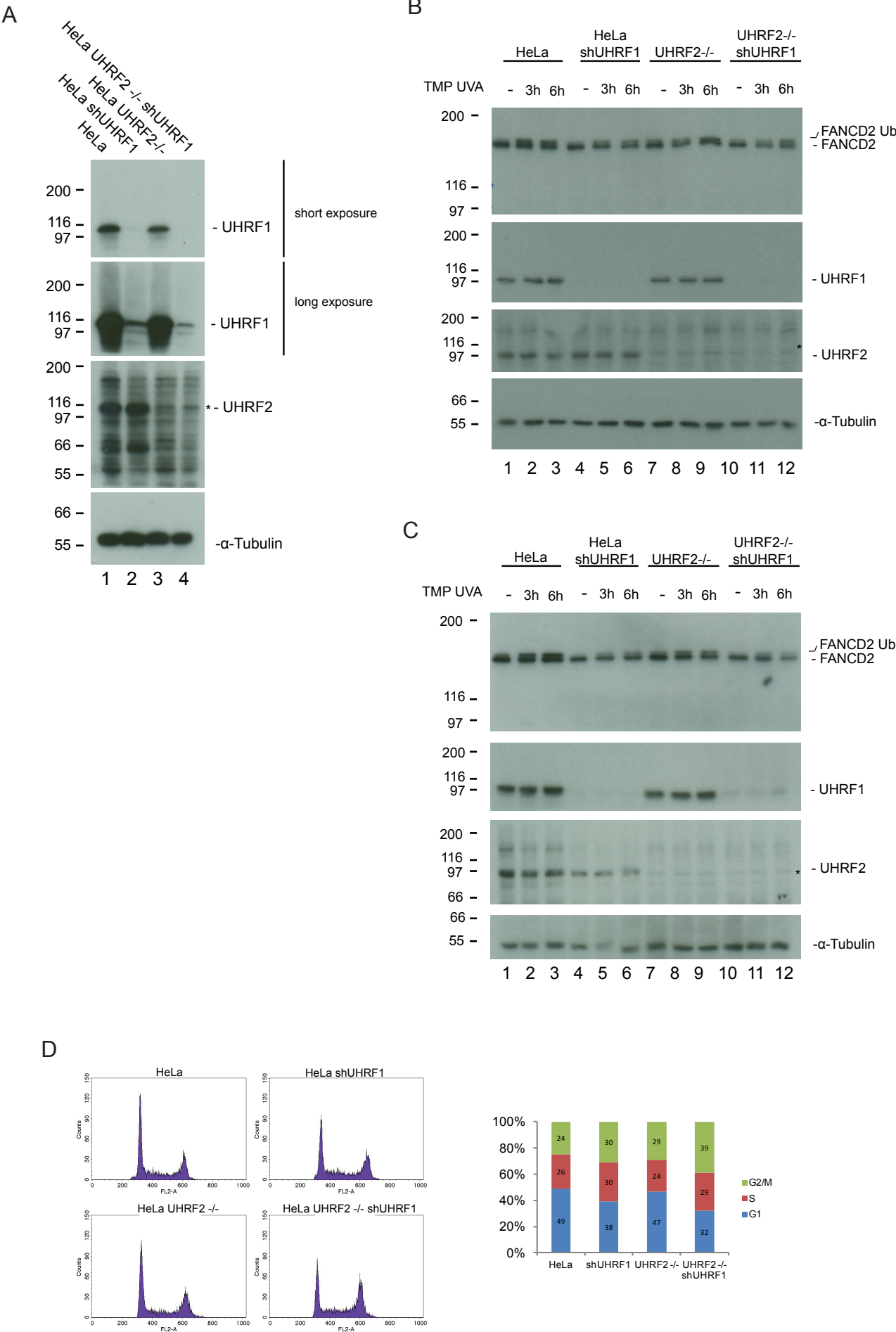

Supplement: S4 Fig — UHRF1 and UHRF2 are required for normal activation and recruitment of FANCD2. A) Western blot analysis of lysates from HeLa cells or HeLa cells where UHRF1 and/or UHRF2 were depleted using shRNA-mediated knockdown or CRISPR/Cas9-mediated knockout. B) and C) Western blot analysis of lysates from HeLa cells or HeLa cells where UHRF1 and/or UHRF2 were depleted using shRNA-mediated knockdown or CRISPR/Cas9-mediated knockout following treatment with TMP/UVA and harvested at 3 and 6 hours. Strong accumulation of monoubiquitinated FANCD2 (FANCD2-Ub) occurs in HeLa cells but is reduced when UHRF1 and UHRF2 are depleted. Replicates used for quantification in Fig 4B. D) FACS analysis of cell lines used in Fig 4B and 4E. Depletion of UHRF1 or UHRF2 does not impact the cell cycle distribution. (PDF) [file pgen.1007643.s004.pdf]

Figure S5

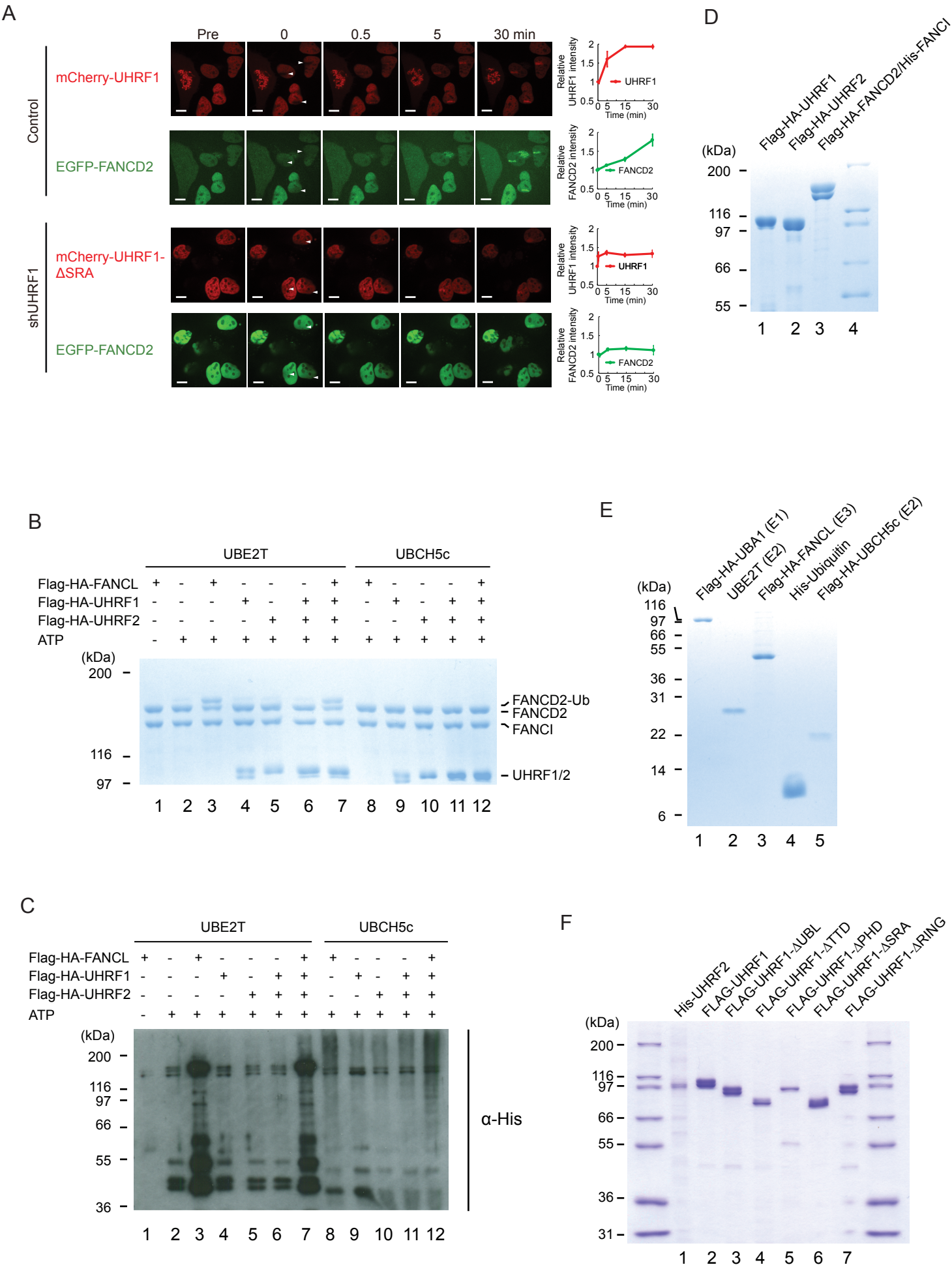

Supplement: S5 Fig — UHRF1 and UHRF2 are not E3 ligases for FANCD2. A) HeLa cells expressing EGFP-tagged FANCD2 and shRNA resistant mCherry-UHRF1 with and without the SRA domain where subjected to depletion of by shRNA, pre-treated with TMP, and then microirradiated at the sites indicated with white arrows. Charts indicate quantification of relative intensity of signal at the irradiated sites. Disruption of the SRA domain greatly reduced both UHRF1 and FANCD2 recruitment. Scale bar indicates 10μm. Error bars show SEM, n = 3/treatment. B) In vitro ubiquitination assay of FANCD2. Individual components were purified from Sf9 insect cells. FANCL (E3 ligase) supports robust ubiquitinatination of FANCD2. Replacement of FANCL by UHRF1 or UHRF2 does not support FANCD2 monoubiquitination. Switching the E2 ligase UBE2T with UBCH5c (C5) does not allow ubiquitination of FANCD2. C) Western blot of ubiquitination reactions in (B) using an anti-His antibody. The ubiquitin in the ubiquitination reaction is His tagged and is visualized. D) and E) Coomassie blue stain of recombinant proteins used in B. F) Coomassie blue stain of recombinant His-UHRF2, and Flag-tagged wild type and deletion mutants of UHRF1 purified from Sf9 cells, which were used in the experiments presented in Fig 4D. (PDF) [file pgen.1007643.s005.pdf]

Figure S6

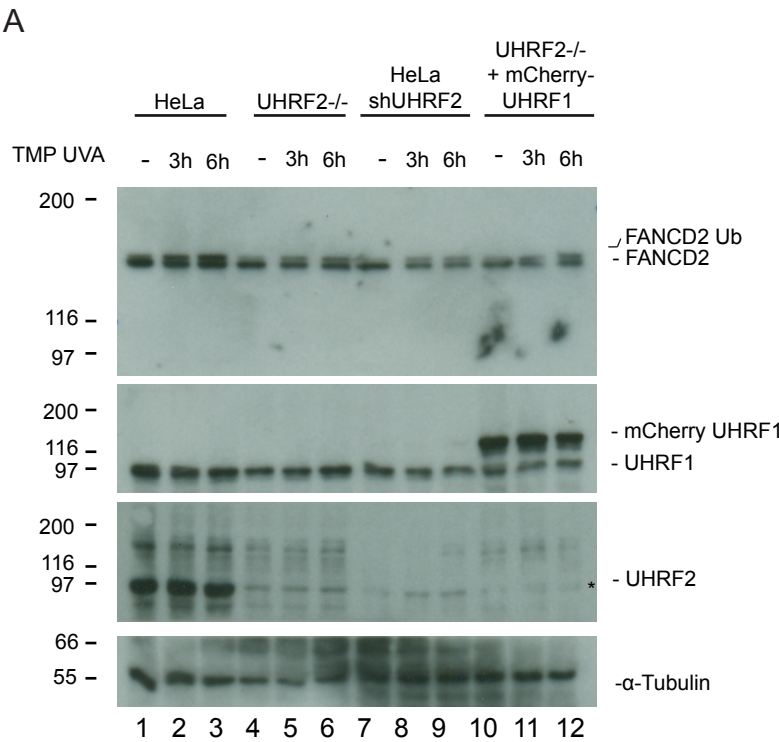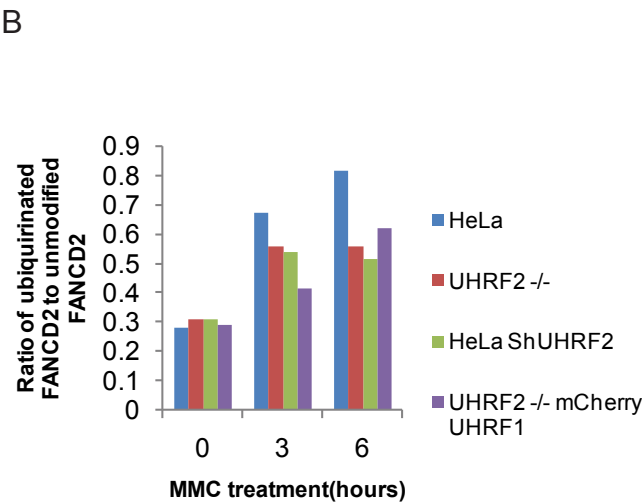

Supplement: S6 Fig — Both UHRF1 and UHRF2 are required for FANCD2 monoubiquitination, and they are not redundant. A) Western blot analysis of lysates from HeLa cells or HeLa cells where UHRF2 was depleted using shRNA-mediated knockdown or CRISPR/Cas9-mediated knockout, or mCherry tagged UHRF1 was expressed exogenously, following treatment with TMP/UVA and harvested at 3 and 6 hours. Strong accumulation of monoubiquitinated FANCD2 (FANCD2-Ub) occurs in HeLa cells but is significantly reduced when UHRF2 is depleted and cannot be rescued by UHRF1 over-expression. Asterisk indicates unspecific band. B) Chart shows ratio of FANCD2-Ub to FANCD2. (PDF) [file pgen.1007643.s006.pdf]

Figure S7

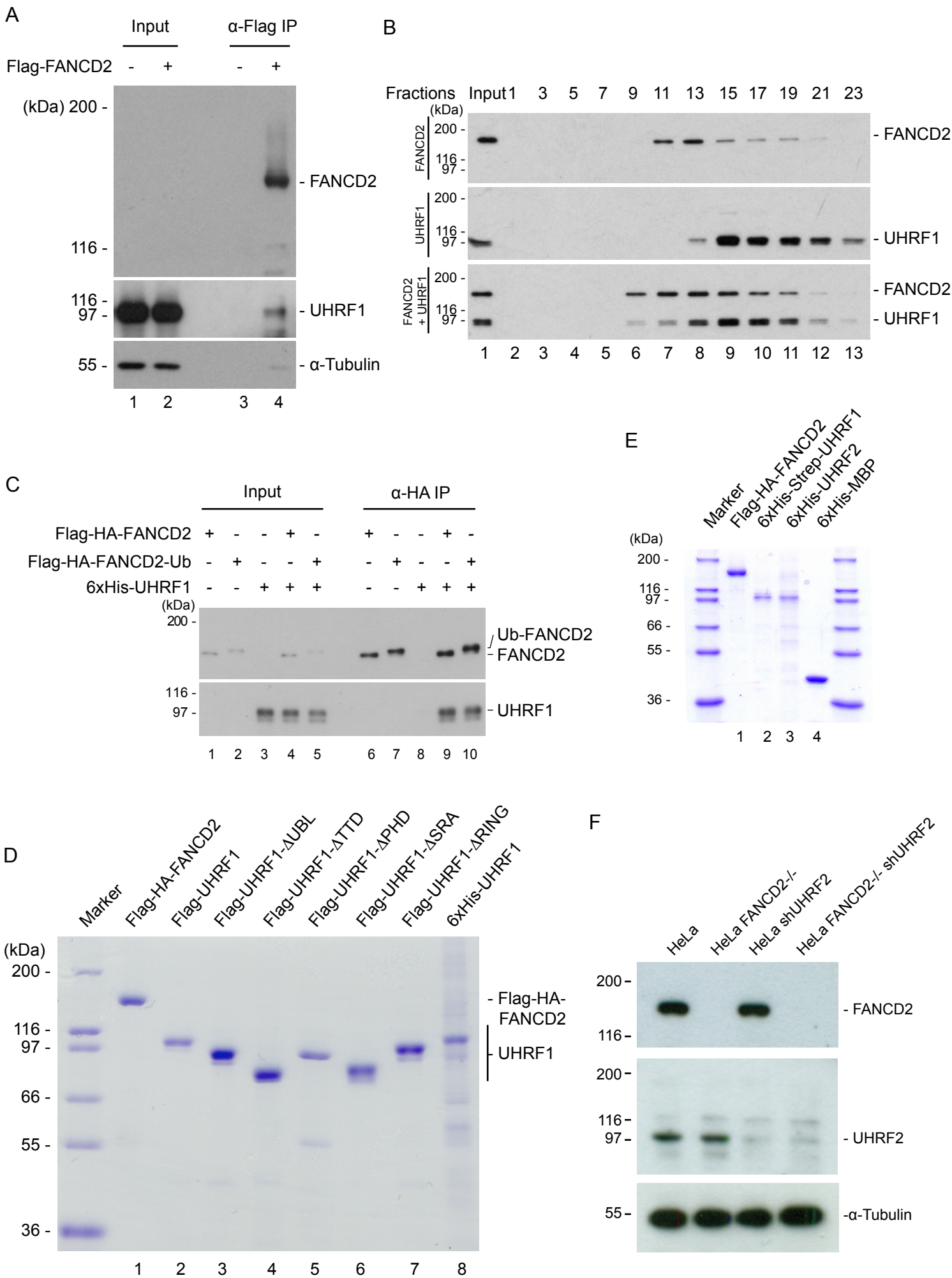

Supplement: S7 Fig — UHRF1 interacts directly with FANCD2. A) Immunoprecipitation of Flag-FANCD2 from HeLaS3 cells where endogenous FANCD2 was depleted by shRNA. HeLaS3 was used as a negative control. Cells were treated with TMP/UVA and allowed to recover for 1 hour before lysis, immunoprecipitation and immunoblotting. B) Size exclusion chromatography of FANCD2 (top), UHRF1 (middle) or FANCD2 incubated with UHRF1 in vitro (bottom). Recombinant proteins were purified from Sf9 cells. Proteins were analyzed on a Superdex 200 5/150 GL chromatography column. C) In vitro binding assay of HA-FANCD2, monoubiquitinated HA-FANCD2 (Ub-HA-FANCD2), both purified from HeLa cells, and 6xHis-UHRF1 purified from Sf9 cells. UHRF1 co-immunoprecipitates equally well with HA-FANCD2 and Ub-HA-FANCD2, showing that FANCD2 interacts with UHRF1 independently of monoubiquitination. D) Coomassie blue stain of recombinant Flag-HA-tagged FANCD2, Flag-tagged wild type and deletion mutants UHRF1 and 6xHis-tagged UHRF1 purified from Sf9 cells, which were used in the experiments presented in Fig 6. E) Coomassie blue stain of recombinant Flag-HA-tagged FANCD2, 6xHis-Strep-tagged UHRF1, and 6xHis-tagged UHRF2 purified from Sf9 cells and 6xHis tagged MBP purified form E. coli, which were used in the experiment presented in Fig 6D. F) Cells used for the clonogenic survival assay experiment shown in Fig 6F were harvested and subjected to immunoblot analysis using the indicated antibodies. (PDF) [file pgen.1007643.s007.pdf]
