# Supplementary material for: Identification of UHRF2 as a novel DNA interstrand crosslink sensor protein
Source: PLoS Genet. 2018 Oct 18;14(10):e1007643. doi: 10.1371/journal.pgen.1007643 (PMC6193622; doi:10.1371/journal.pgen.1007643)
Supplement: S1 Table — (PDF) [file pgen.1007643.s008.pdf]

Compound	Adduct	Precursor Ion (*m/z*)	MS1 Resolution	Product Ion (*m/z*)	MS2 Resolution	Retention Time (min)	Dwell Time (ms)	CE (V)	CA (V)	Polarity
dC	dC-H$^+$	228	Unit	112	Unit	3.5	500	10	4	Positive
	dC-Na$^+$	250	Unit	134	Unit	3.5	500	10	4	Positive
5hmdC	hmdC-H$^+$	258	Unit	142	Unit	4.1	500	12	4	Positive
	hmdC-Na$^+$	280	Unit	164	Unit	4.1	500	12	4	Positive
5mdC	mdC-H$^+$	242	Unit	126	Unit	8.7	500	10	4	Positive
	mdC-Na$^+$	264	Unit	148	Unit	8.7	500	10	4	Positive
dG	dG-H$^+$	268	Unit	152	Unit	9.4	500	10	4	Positive
	dG-Na$^+$	290	Unit	174	Unit	9.4	500	10	4	Positive
T	dT-H$^+$	243	Unit	127	Unit	10.9	500	10	4	Positive
	dT-Na$^+$	265	Unit	149	Unit	10.9	500	10	4	Positive
dA	dA-H$^+$	252	Unit	136	Unit	13.4	500	10	4	Positive
	dA-Na$^+$	274	Unit	158	Unit	13.4	500	10	4	Positive
